# Episodic Future Thinking in Semantic Dementia: A Cognitive and fMRI Study

**DOI:** 10.1371/journal.pone.0111046

**Published:** 2014-10-21

**Authors:** Armelle Viard, Pascale Piolino, Serge Belliard, Vincent de La Sayette, Béatrice Desgranges, Francis Eustache

**Affiliations:** 1 Inserm, U1077, Caen, France; 2 Université de Caen Basse-Normandie, UMR-S1077, Caen, France; 3 Ecole Pratique des Hautes Etudes, UMR-S1077, Caen, France; 4 Centre Hospitalier Universitaire, U1077, Caen, France; 5 Université Paris Descartes, Institut de Psychologie, Memory and Cognition Lab, Paris, France; 6 Inserm UMR S894, Centre de Psychiatrie et Neurosciences, Paris, France; 7 CHU Pontchaillou, Rennes, France; INSERM U894, Centre de Psychiatrie et Neurosciences, Hopital Sainte-Anne and Université Paris 5, France

## Abstract

Semantic dementia (SD) is characterized by gradual loss of semantic memory. While episodic autobiographical memory seems relatively preserved, behavioral studies suggest that episodic future thinking is impaired. We used fMRI to measure brain activity in four SD patients (JPL, EP, LL, EG) while they envisioned future events and remembered personal past events. Twelve healthy elders served as controls. Episodic quality, emotion, mental imagery and level of consciousness (via remember/know judgements) were checked at debriefing. We analyzed the future compared to the past for each patient. All patients presented lateral temporal atrophy, but varied in terms of frontal and anterior hippocampal atrophy. Patient JPL presented atrophy in bilateral superior medial frontal gyri and left anterior hippocampus and was unable to engage in episodic future thinking, despite hyperactivations in frontal and occipital regions. Patient EP presented no atrophy in the anterior hippocampus, but atrophy in bilateral superior medial frontal gyrus and had difficulties to engage in episodic future thinking. Patient LL presented atrophy in left anterior hippocampus, but hyperactivated its right counterpart for future compared to past thinking, permitting her to project efficiently in the future in an episodic way. Patient EG presented no atrophy in the superior medial frontal gyri or anterior hippocampi and was able to engage in episodic future thinking. Altogether, patients' future projections differed depending on the severity and localization of their atrophy. The functional integrity of bilateral superior medial frontal gyri and anterior hippocampus appear crucial for episodic future thinking: atrophy of both structures strongly impairs future projection, while integrity of these structures or hyperactivation of residual tissue normalizes episodic future projection.

## Introduction

Semantic dementia (SD) is a variant of fronto-temporal dementia characterized by a gradual loss of semantic memory [1], with progressive anomia and deterioration of vocabulary [2]). An asymmetrical atrophy of the lateral temporal lobe is generally observed [3] with an antero-posterior gradient, the highest changes located in its anterior portion [4]. A relative preservation of episodic memory is observed [5], [6], with intact recent and day-to-day memory. Episodic memory is characterized by a particular self-reflective mental state, termed autonoetic consciousness, which implies that a person recollects his/her personal events with a sense of re-experiencing, by mentally “travelling in time” in the past [7], [8]. Converging lines of evidence from different fields of research indicate that remembering the past or envisioning the future share common cognitive [9], [10], [11] and neural underpinnings [12], [13], [14], [15]. Indeed, past and future thinking build on similar information stored in episodic memory and rely on similar cognitive processes (i.e., self-referential processing, imagery and flexible recombination of stored details). Yet, in semantic dementia, behavioral studies have consistently shown that future thinking is impaired [16], [17], [18].

Duval et al. [18] examined self-representations and states of consciousness (measured via remember/know judgements) in SD patients for past, present and future periods. The future period elicited poorer performances than the past and present periods in terms of episodic self-representations and autonoetic consciousness. SD patients had difficulties in projecting themselves into the future and had an impaired level of consciousness associated to the future. These findings suggest that personal semantic memory is necessary to imagine one's future self, as confirmed by Irish et al. [16]. Indeed, Irish et al. [16] also indicated that SD patients had difficulties in episodic future simulation, despite a relative preservation of past episodic retrieval. They showed that SD patients' future thinking deficit was driven by their difficulty in providing episodic (specific) details, contrasting with elevated semantic (factual) details. SD patients' phenomenological past experiencing however (measured by subjective ratings of vividness, valence, emotional intensity, personal significance) did not differ from controls. This reveals a disconnect between objective task performances and subjective phenomenological pre-experiencing when generating future events in this population. Irish et al. [17] showed relatively intact episodic retrieval but significant impairments for episodic future thinking in their group of SD patients. Atrophy in areas of the lateral temporal cortex (left inferior temporal gyrus and bilateral temporal pole) was found to correlate with deficits in episodic future thinking. This region is known for its role in semantic processing and thus confirms previous findings that semantic knowledge is critical for the construction of novel future events [19].

Atrophy in the lateral temporal cortex is characteristic of semantic dementia and as indicated above may play a role in SD's episodic future thinking deficit, although other regions are also important (e.g., hippocampus, medial prefrontal cortex). Evidence of hippocampal atrophy is now well documented in SD, even in the early stages of the disease [20], [21], [22], [23], [24], [25], [26], [4], [27], [28], [29], [6], although some patients were reported to have no hippocampal atrophy [30], [6]. The hippocampus supports relational processing [31], [32], [33], including flexible recombination of details for past and future event construction [34]. Addis and Schacter [35] showed that future-associated activity in the anterior hippocampus was associated with higher demands on recombination of details. Hence, atrophy in this region may strongly affect episodic future thinking. In joint collaboration with the hippocampus, the medial prefrontal cortex is also critical in episodic future thinking. Its anterior part seems more involved in information integration and self-referential thinking, while its dorsal part has a role in generative construction [36].

Studies on future thinking in SD are scarce and no fMRI study is yet available. Neuroimaging studies on future thinking in healthy adults have shown a neural network which has striking similarities with the one recruited during episodic past remembering, including prefrontal and medial temporal cortices, medial parietal (posterior cingulate and retrosplenial cortices), posterior parietal (precuneus and temporo-parietal junction), occipital regions and the cerebellum [12], [13], [14], [15], [37], [38]. We previously showed that a common network of brain regions was activated for past and future thinking in healthy older adults, reflecting the use of similar cognitive processes [19]. Our results also showed that the episodic nature of future events depended on the inferior frontal and lateral temporal gyri, involved in semantic memory retrieval.

Here, we examined episodic future thinking in four SD patients using fMRI and compared it to past remembering. A group of 12 healthy elders served as a comparison group. In the scanner, upon presentation of a cue-phrase prompting a specific past or future event (obtained by questioning a close family member), patients were asked to mentally recall specific events from the past 12 months and specific plans they intended to pursue in the next 12 months. We also collected several behavioral data to assess the degree of phenomenological (re/pre)experiencing, through ratings of episodic quality, emotional intensity, valence, mental visual imagery, level of consciousness and repetition. Beyond lateral temporal atrophy which was present in all patients and may impact episodic future thinking, each had atrophy in different regions of the episodic future thinking network. We predicted that anterior hippocampal and/or medial prefrontal atrophy would strongly impair episodic future thinking given the crucial role these regions play. We expected that, in the presence of atrophied regions within this network, different processes might arise: either activation in residual tissue, up-regulation of areas within the network or recruitment of additional brain areas.

## Materials and Methods

### Participants

#### Patient details

We studied four patients suffering from SD selected according to a codified procedure in French qualified centres by senior neurologists (VDLS & SB) whose major activity is dedicated to the diagnosis and follow-up of patients suffering from neurodegenerative disorders including semantic dementia, in addition to a neuropsychologist and a speech therapist. The four patients all presented the two core features of SD (i.e., impaired picture naming and single-word comprehension) and the three following additional criteria, as specified by Gorno-Tempini et al. [39]: impaired object knowledge, spared repetition, spared speech production (i.e., fluent) and/or surface dyslexia and severe anterior temporal atrophy (see VBM analyses). The clinical and neuropsychological follow-up of our patients, who have been re-examined between 5 to 8 years after their first consultation, has confirmed the initial diagnosis (i.e., predominant semantic memory deficits, and preserved spatial orientation and autonomy). Table 1 summarizes patients' main neuropsychological scores and z-scores. Of note, all participants were scanned within the same week as neuropsychological testing.

**Table 1:** Scores on neuropsychological tests for patients JPL, EP, LL, and EG.

Neuropsychological tests	HE (mean ± s.d.)	JPL	z-score	EP	z-score	LL	z-score	EG	z-score
*Episodic memory*									
Grober and Buschke									
Immediate recall (/16)	15.42±0.99	**12**	**−3.455**	16	0.586	16	0.586	16	0.586
Free recall (/48)	31.75±5.36	24	−1.446	31	−0.140	29	−0.513	30	−0.326
Total recall (/48)	46.67±1.92	**38**	**−4.516**	47	0.172	48	0.693	47	0.172
Delayed recall (/16)	11±2.22	9	−0.901	14	1.351	**6**	**−2.252**	8	−1.351
Rey complex figure									
Copy (sec)	33.90±2.44	36	0.875	36	0.875	36	0.875	33	−0.375
Recall (sec)	16.93±7.33	25	1.101	18	0.146	10.5	−0.877	20	0.419
*Semantic memory*									
Fluency tasks									
Category fluency (/2 min)	32.64+/−7.9	**17**	**−1.980**	**9**	**−2.992**	**16**	**−2.106**	**9**	**−2.992**
Letter fluency (/2 min)	23.78+/−8.35	**7**	**−2.010**	26	0.266	**8**	**−1.890**	**5**	**−2.249**
Semantic Knowledge Task									
Picture naming (/36)	36+/−0.0	**30**	**pathological**	**32**	**pathological**	**35**	**pathological**	NA	-
Superordinate category (/54)	54+/−0.0	54	OK	54	OK	54	OK	NA	-
Specific attributes (/54)	54+/−0.0	**49**	**pathological**	**50**	**pathological**	**50**	**pathological**	NA	-
BECS-GRECO Semantic Battery									
Words/40)	39.4+/−0.9	NA	-	NA	-	NA	-	**22**	**−19.33**
Pictures (/40)	39.2+/−0.9	NA	-	NA	-	NA	-	**37**	**−2.44**
French celebrities questionnaire (%)	100+/−3.0	**39.1**	**−19.96**		NA	**34.8**	**−21.4**	**2.5**	**−31.17**
Mill Hill (/33)	25.64±5.84	**16**	**−1.651**	29	OK	20	OK	29	OK
*Language*									
Speech production	NA	Fluent		Fluent		Fluent		Fluent	
Repetition	NA	OK		OK		OK		OK	
Reading	NA	OK		OK		OK		OK	
LEXIS battery									
Picture naming (/80)	72.8±3.28	**55**	**−5.427**	**60**	**−3.902**	NA	-	**47**	**−7.866**
Picture naming (/64)	58.5±3.21	NA	-	NA	-	**47**	**−3.583**	NA	-
*Executive functions*									
TMT B-A (sec)	76±64	30	−0.719	35	−0.641	63	−0.203	66	−0.156
*Other*									
MMSE (/30)	28.42±1.38	27	−1.029	29	0.420	**25**	**−2.478**	28	−0.304
GDS (/30)	4.83±3.04	7	0.714	5	0.056	9	1.372	7	0.714
Mattis (/144)	142.17±1.34	**132**	**−7.590**	144	1.366	141	−0.873	**120**	**−16.545**

*Tests*: BECS GRECO Semantic Battery: Merck et al. (2011); French celebrities questionnaire: Belliard et al. (2001); GDS: Yesavage et al. (1983); Grober and Buschke (1987); Mill Hill test: Deltour (1993); Mattis: dementia rating scale (Mattis, 1976); MMSE: Mini-Mental Status Exam (Folstein et al., 1975); Rey complex figure: Rey (1970); Semantic Knowledge Task: Desgranges et al. (1996); Picture naming: LEXIS (de Partz, 2001); TMT: Trail Making Test (Reitan, 1958).

*Note*: The Semantic Knowledge Task was proposed to patients from Caen (JPL, EP, LL); the BECS GRECO Semantic Battery was proposed to the patient from Rennes (EG). On the Semantic Knowledge Task, performances had to be maximum to be considered unimpaired (i.e., standard deviation in control group equals zero). Hence, if patients did not obtain the maximum score, their performance was impaired (noted “pathological” in the Table). The French celebrities questionnaire was not proposed to EP at the time she participated in this fMRI study.

Results were considered as significant when z>±1.96, p<0.05. Pathological scores appear in bold and when no standard deviation was available, pathological performances were noted “pathological” in bold. HE = healthy elders; OK = performance not significantly different from healthy elders; NA = not available.

JPL, a 62-year-old right-handed male retired cutter with 10 years of formal education, was seen at the University Hospital Center of Caen in November 2006. JPL reported progressive memory loss, particularly involving remembering peoples' names or familiar places, difficulty in word-finding and impaired object knowledge (e.g., he would eat an orange without pealing it). His wife reported a tendency to irritability, lack of initiative and poor conversational interactions, but his day-to-day memory and spatial orientation were preserved. As a leisure activity, he played sudoku. On formal neuropsychological testing, JPL showed relatively well preserved episodic memory (good free recall, but impaired immediate recall) as measured by a procedure derived from the Grober and Buschke test [40] (see Table S9 in File S1 for brief explanation of this test and what it measures). Visuospatial abilities were good as measured by copying and delayed recall of the Rey complex figure [41]. He scored poorly on semantic memory tests, as measured by category (names of animals) and letter (words beginning by letter p) fluency tasks [42] (see Table S9 in File S1 for brief explanation of this test and what it measures). On the Semantic Knowledge Task [43] (see Table S9 in File S1 for brief explanation of this test and what it measures), he was impaired for picture naming and object knowledge, as shown by his difficulty in providing specific attributes of words. He was impaired at recognizing famous faces on the French Celebrities Questionnaire [44] (see Table S9 in File S1 for brief explanation of this test and what it measures). He performed poorly on the Mill Hill test [45], a multiple-choice synonym vocabulary questionnaire which assesses verbal knowledge. His language abilities were tested with the LEXIS battery [46] (see Table S9 in File S1 for brief explanation of this test and what it measures). He produced semantic paraphasias and presented difficulties in picture naming, word-finding, word definitions (e.g., watermelon, deer, hammock, city hall…) and single-word comprehension (e.g., when asked “what is the capital of France?”, he would answer: “what does capital mean?” or when asked “in which country is Venice?”, he would answer, “what is Venice?”). His speech production was fluent. Repetition, reading of a text and its restitution were spared. He presented no deficit on the Trail Making Test [47].

EP, a 77-year-old right-handed female retired general practitioner with 20 years of formal education, was seen at the University Hospital Center of Caen in April 2008. EP reported difficulties in word-finding and word comprehension, especially animal names and geographic locations which she once knew well. She had impaired object knowledge (e.g., for EP, zebras live in French prairies; salads and carrots grow in forests). She continued to have stimulating intellectual activities by reading and attending university senior courses, although she complained not being able to learn efficiently. She was autonomous in daily life and her day-to-day memory was preserved. On formal neuropsychological testing, EP showed relatively well preserved episodic memory on the Grober and Buschke test. Visuospatial abilities were good as measured by copying and delayed recall of the Rey complex figure. Given her high level of education, she scored poorly on semantic memory, as measured by category fluency tasks (names of animals). She produced errors on picture naming and on object knowledge, as shown by her difficulty in providing specific attributes of words on the Semantic Knowledge Task. She performed well on the Mill Hill vocabulary test. Her language abilities, tested with the LEXIS battery, confirmed her difficulties in picture naming. She produced paraphasias (semantic, visual, visuo-semantic), had difficulties on single-word comprehension and in providing word definitions (zebra, hippopotamus, elephant, kiwi…), with many specific attributes being lost. Her speech production was fluent. Repetition and reading were spared. She presented no deficit on the TMT.

LL, a 73-year-old right-handed female retired secretary with 9 years of formal education, was seen at the University Hospital Center of Caen in September 2007. LL reported difficulties in word-finding, object knowledge (e.g., she will cook cucumber and radish; she will put sugar on avocados) and recognizing people. She was fond of logical games (e.g., sudoku). She was autonomous in daily life and her day-to-day memory was preserved. On formal neuropsychological testing, LL had preserved episodic memory (on immediate and free recalls) on the Grober and Buschke test. Visuospatial abilities were good as measured by copying and delayed recall of the Rey complex figure. She scored poorly on semantic memory tests, both on category (names of animals) and letter (words beginning by letter p) fluency tasks. She produced errors on picture naming and on object knowledge (difficulty in providing specific attributes of words) on the Semantic Knowledge Task. She was impaired at recognizing famous faces on the French Celebrities Questionnaire. She performed well on the Mill Hill vocabulary test. Her language abilities, tested with the LEXIS battery confirmed her difficulties in picture naming. She also showed difficulties on single-word comprehension and made errors on word definitions (e.g., bull, kangourou, harp, volcano…). Her speech production was fluent. Repetition, reading of a text and its restitution were spared. She presented no deficit on the TMT.

EG, a 62-year-old right-handed female retired secretary with 9 years of formal education, was seen at the University Hospital Center of Rennes in October 2008. EG reported difficulties in word-finding, names of places and recognizing people. She also showed oral and written comprehension difficulties. Her spatial orientation was good and she mentioned that she frequently consulted atlases to resituate her trip itineraries. She was autonomous in daily life and her day-to-day memory was preserved. Her leisure activities consisted in playing with logical games (e.g., sudoku, video games). On formal neuropsychological testing, EG showed preserved episodic memory (on immediate, free and delayed recalls) on the Grober and Buschke test. Visuospatial abilities were good as measured by copying and delayed recall of the Rey complex figure. She scored poorly on semantic memory tests, both on category (names of animals) and letter (words beginning by letter p) fluency tasks. Being a patient from Rennes (not Caen, like the three other patients), clinicians there proposed the BECS GRECO Semantic Battery [48] to EG, instead of the Semantic Knowledge Task. On the BECS GRECO Semantic Battery, she was impaired on single-word comprehension (difficulty on semantic matching of words) and on object knowledge (difficulty on semantic matching of pictures). She was extremely impaired at recognizing famous faces on the French Celebrities Questionnaire. She presented surface dyslexia (e.g., baptism; note that in French, the [p] is not pronounced). She performed well on the Mill Hill vocabulary test. Her language abilities, tested with the LEXIS battery), confirmed her difficulties in picture naming. Her speech production was fluent. Repetition and reading were spared. She presented no deficit on the TMT.

For all patients, an indicator of disease severity was obtained with the Mini-Mental Status Exam [49] (MMSE; total score/30) which assesses both comprehension and expression abilities through eight different questions. The MMSE has already been used as an indicator of disease severity. Matuszewski et al. [50] subdivided their two groups of SD patients into a mild group (mean score on the MMSE of 26±1.91) and a moderate group (mean score of 21.29±1.07). Based on the MMSE (see Table 1), the four patients (JPL = 27; EP = 29; LL = 25; EG = 28) show high scores, indicative of an early stage of dementia (i.e., mild stage). Note that all patients were autonomous in daily life. Global cognitive functioning was assessed via the Mattis dementia rating scale [51] (total score/144; see Table S9 in File S1 for brief explanation of this test and what it measures). Signs of depression were estimated with the Geriatric Depression Scale [52] (GDS; total score/30). Table 1 summarizes patients' neuropsychological scores and z-scores according to normative data provided for each test.

#### Healthy elders

Twelve right-handed healthy elders (females, mean age ± s.d. = 67.2±5.2 years; ranging from 60 to 75 years old), mean years of education (11.42±2.81), with no history of psychiatric or neurological disorder were recruited through a senior university, a retirement association or a newspaper advertisement. Participants had no abnormality on their T_1_-weighted high-resolution magnetic resonance imaging (MRI). They underwent a battery of neuropsychological tests to assess their cognitive abilities and all performed in the normal range (see Viard et al. [53] for a full description). Each participant resided at home and all were active in cultural pursuits, continuing education or with responsibilities in diverse associations.

The study was approved by the Regional Ethics Committee of Lower Normandy and written informed consent was obtained from all participants prior to their participation in the study. Patients understood the experiment and had full capacity to consent (i.e., no surrogate consent procedure was needed). Data on healthy elders were obtained as part of a broader experiment exploring five past periods previously published [53], [54] and a future period [19]. These healthy subjects were scanned with the intention to be compared with the patients. Results of two SD patients (JPL and EP) on a task exploring episodic autobiographical memory retrieval over five past periods have been published previously [6]. Here, we present new results concerning four SD patients on the episodic future thinking task and compare them to the group of healthy elders. Of note, given the rarity of patients with semantic dementia, we were not able to perfectly match each patient to the control group. This group of healthy elders can nonetheless serve as a comparison group to our patients, given that disparities have been controlled for by adding covariates (age and years of education) in statistical analyses (see below).

### Task and experimental design

Autobiographical memories from the last 12 months and future plans scheduled in the next 12 months were obtained through an interview with a close family member a week before the scanning session. The family member, in most cases the spouse, was close enough to provide specific personal past memories of the participant and actual future plans that the participant intended to pursue (i.e., events were not made up by family members, but corresponded to actual past or future events in the participant's life). Among events provided by the family member, only those which corresponded to specific (i.e., episodic) events, unique in time and space, were selected. Before scanning, participants were familiarized with the task in a training phase outside of the scanner, using different events than in the experimental task. Special care was taken to explain to the patients the tasks they had to perform. The instructions given to participants for the past period were as follows: “You will see on the screen short sentences which refer to specific past events which happened to you in the last 12 months, followed by a black screen. Press on the button as soon as you can access a specific personal past event, which lasted less than 24 hours, related to the cue and think about all the details that come to mind, until the cross appears. Try to remember the event as if you were reliving it.” The instructions given to participants for the future period were as follows: “You will see on the screen short sentences which refer to future plans which you intend to pursue in the next 12 months, followed by a black screen. Press on the button as soon as you can access a specific personal future event, which will last less than 24 hours, related to the cue and think about all the details that come to mind, until the cross appears. Try to envision the event as if you were living it in advance.” In the scanner, personal sentence-cues were presented visually in white on a black background, using Superlab software (3.0 version, Cedrus). During the whole experiment, participants were asked to keep their eyes open to be able to read the sentence cues. Upon presentation of the visual cue, participants were instructed to recall or envision a specific detailed event, unique in time and space, that had either occurred in the past 12 months (past period) or was scheduled in the next 12 months (future period). For both past and future events, they were asked to experience the event with as much details as possible. Past and future events were sufficiently different to refrain participants from simply recalling a similar past event when instructed to envision a future event. Two functional runs, one per period, each lasting 5 minutes, were composed of 5 experimental (i.e., 5 events) and 5 control blocks, randomly intermixed across subjects. In the experimental condition, one block consisted of a sentence-cue (5 s) followed by a blank screen (19 s) during which participants had to mentally experience the corresponding specific personal event (e.g., past: “my grandson's last birthday party”; future: “my fifty-third wedding anniversary”). In total, they had to recall five episodic past events and envision five episodic future events. They were asked to press on a button as soon as they gained access to the prompted event. In the control condition, participants were asked to detect the presence of two consecutive letters (“mb”) in pseudo-words of six letters (for example, “speugr” or “mbieha”) and were instructed to press on a button when “mb” was present in the pseudo-word. Five pseudowords were presented in each control block, each lasting 24 seconds (1 second for cue presentation, followed by 3.8 seconds for the response). All participants were scanned on the same scanner (1.5 tesla) at the University Hospital Center of Caen.

Following the scanning session, the debriefing took place (mean duration = 2,5 hours) in order to verify participants' engagement in the tasks and to identify the nature of the evocations experienced during scanning. Participants were asked to retrieve the five past and five future events again, using the same sentence-cues as in the scanner, but this time aloud in order to check that the events corresponded to the expected cued events and to assess their episodic nature (i.e., specificity and detail). Their episodic nature (or “episodicity”) was estimated 1) with “objective” measures, using a 5-point scale (generating overall autobiographical and strictly episodic scores; see below) and 2) with “subjective” measures, by collecting participants' ratings on several behavioral scales (evaluating emotion, mental imagery, level of consciousness and effect of repetition; see below).

First, the specificity of each evocation was measured by the investigators (objective measurement of episodicity) using a fine-grained 5-point scale (0-1-2-3-4) similar to previous episodic scales used with healthy subjects and patients with cerebral diseases [55], [56], [57], [58], [59], [50], [53], [19], [6]. This scale takes into account the specificity of the content (single or repeated event), the spatiotemporal situation and the presence of details (perceptions, thoughts, feelings). A specific event, situated in time and space, with sensory details is given a score of 4. A specific event with few details, but situated in time and space, scores 3. A generic event (repeated or prolonged over time, situated in time and space) scores 2. A vague event (repeated or prolonged over time, not situated in time and space) scores 1. Absence of memory, or general information about a theme, scores 0 (see Table S10 in File S1). Two different total scores are obtained per period. First, the overall autobiographical score (AS: maximum score per period 4×5 = 20) includes all the events (specific and generic) and corresponds to the classic episodic memory score used in the well-known Autobiographical Memory Interview [56] (AMI). The AS score is expressed in terms of ratio (i.e., scores obtained for each of the five events per period were summed and divided by the maximum score per period which is 20). Second, the strictly episodic score (ES: maximum score per period 4×5 = 20) includes only the number of specific and detailed events scoring 4, using a more stringent criterion. ES is expressed in terms of ratio of strictly episodic events per period (i.e., number of strictly episodic events divided by the number of retrieved events). Two independent experts rated each event recalled at debriefing and any difference of opinion between them was discussed until a consensus was reached. A final score was calculated based on the number of episodic (past or future) events experienced by participants in the scanner (e.g., since EP experienced one episodic future event, her score was of one for the future; see Table 2).

**Table 2:** Behavioral ratings for patients JPL, EP, LL, EG and healthy elders (HE) and t values comparing each patient to the control group for each period.

	HE		JPL		EP		LL		EG	
	Past	Future	Past	Future	Past	Future	Past	Future	Past	Future
***Episodic scores***										
***AS score***										
Mean ± sd	3.73 (±0.41)	3.17 (±0.57)	3.00	1.20	3.60	2.40	3.80	3.60	3.40	2.60
T			−1.71	**−3.32**	−0.31	−1.29	0.16	0.73	−0.77	−0.96
***ES score***										
Mean ± sd	3.20 (±1.08)	1.73 (±1.31)	0.80	0.00	2.40	0.80	3.20	2.40	2.40	0.00
T			−2.14	−1.27	−0.71	−0.68	0.00	0.49	−0.71	−1.27
***Nb episodic events***										
Mean ± sd	4.75 (±0.45)	4.00 (±1.16)	4.00	1.00	5.00	1.00	5.00	4.00	4.00	3.00
t			−1.59	−2.48	0.53	−2.48	0.53	0.00	−1.59	−0.83
***Emotion***										
***Intensity***										
Mean ± sd	5.10 (±1.59)	4.80 (±1.72)	8.73	NA	9.50	9.50	2.72	2.66	5.65	2.15
T			2.20	-	**2.66**	**2.63**	−1.44	−1.20	0.33	−1.48
***Valence***										
Mean ± sd	6.57 (±1.26)	6.75 (±1.05)	8.88	NA	7.80	9.00	6.32	5.70	7.90	4.20
t			1.76	-	0.94	2.06	−0.19	−0.96	1.02	−2.34
***Imagery***										
***Mental strategy***										
Mean ± sd	8.65 (±1.83)	8.86 (±1.69)	8.14	NA	10.00	10.00	9.26	5.54	9.30	6.93
t			−0.27	-	0.71	0.65	0.32	−1.89	0.34	−1.10
***Image number***										
Mean ± sd	5.06 (±2.12)	3.72 (±1.49)	0.80	NA	10.00	10.00	4.40	2.40	1.75	1.50
T			−1.93	-	2.24	**4.06**	−0.30	−0.85	−1.50	−1.43
***Image quality***										
Mean ± sd	9.13 (±1.25)	8.57 (±1.69)	9.10	NA	9.50	5.00	9.28	6.14	8.25	6.95
T			−0.02	-	0.28	−2.03	0.12	−1.38	−0.68	−0.92
***Perspective***										
Mean ± sd	1.40 (±0.59)	1.22 (±0.74)	0.42	NA	1.00	1.00	1.00	1.20	2.00	0.00
t			−1.60	-	−0.65	−0.29	−0.65	−0.03	0.98	−1.59
***Consciousness***										
***R/K***										
Mean ± sd	9.38 (±0.61)	6.28 (±3.47)	9.56	NA	9.90	5.00	9.24	3.22	9.23	4.15
t			0.28	-	0.82	−0.35	−0.22	−0.85	−0.24	−0.59
***Repetition***										
***Rehearsal frequency***										
Mean ± sd	5.51 (±1.99)	4.39 (±2.02)	5.82	NA	0.00	9.50	6.32	4.38	3.10	3.90
t			0.15	-	**−2.66**	2.43	0.39	0.00	−1.17	−0.23
***Last recall***										
Mean ± sd	1.61 (±0.89)	1.46 (±1.49)	0.48	NA	0.50	1.00	2.82	1.82	3.93	4.18
t			−1.22	-	−1.20	−0.30	1.31	0.23	2.50	1.75

*Note:* Behavioral scales range from 0 to 10 for emotional intensity (0 = no emotion to 10 = very strong emotion), emotional valence (0 = very negative to 10 = very positive), mental strategy used (0 = verbal to 10 = visual), number of mental visual images (0 = no images to 10 = over 10 images), mental visual image quality (0 = very blurry to 10 = very clear, state of consciousness (0 = knowing to 10 = remembering), rehearsal frequency (0 = never to 10 = very frequent), last recall (0 = today to 10 = over 10 years ago). For the perspective, the maximum score is 2 (0 = observer; 1 = field and observer; 2 = field).

Results were considered as significant (in bold) when t>±2.576, p<0.01. AS = autobiographical score; ES = strictly episodic autobiographical score; NA = not available; Nb = number; sd = standard deviation; t = t statistic.

Second, subjective measures of episodicity were used to specify the different aspects of the recollective experience. Participants rated their own evocations on several behavioral scales (10-cm lines; self-ratings) known to be crucial to control the degree of episodic (re/pre)experiencing. Emotion is an important phenomenological characteristic of vivid and persistent personal events [60]. Thus, participants were asked to rate their evocations on scales measuring emotional intensity (0 = no emotion to 10 = very strong emotion) and valence (0 = very negative to 10 = very positive). Visual mental imagery is known to increase the recall of specific details [61], [62], [63]. Several scales were used to measure mental visual imagery: mental strategy used (0 = verbal to 10 = visual), number of images (0 = no images to 10 = over 10 images), image quality (0 = very blurry to 10 = very clear) and perspective of mental images [64] (“field” or “observer” perspectives). Regarding perspective, three choices were proposed: observer (0), field and observer (1) or field (2). The autonoetic and noetic levels of consciousness, which characterize episodic and semantic memory respectively, can be distinguished by the remember or know (R/K) paradigm. Participants were asked to rate their evocations on a scale measuring the level of consciousness between the autonoetic and noetic states (scale range from 0 = knowing to 10 = remembering). Unlike the “knowing” state, the “remembering” state is characterized by phenomenal elements associated with (re/pre)experiencing of specific events (visual images, sensations, feelings). For the future, a “Remember” response indicates that the participant was able to pre-experience future events in advance. Recent reactivation (i.e., was an event evoked recently or not) was evaluated using two scales: (1) the frequency of rehearsal scale (0 = never to 10 = very frequent), to determine how frequently an event was rehearsed, prior to scanning and (2) the last recall scale (0 = today to 10 = over 10 years ago), to determine when each event was last recalled. Unfortunately, for JPL, subjective rating scales for the future are not available due to lost material. However, the subjective rating scales for the past and objective measures for both periods are available.

### MRI data acquisition

A blocked functional MRI design was used. Lying in the scanner, participants viewed the display via a mirror to an active matrix video projector. Stimulus onset was synchronized with the acquisition of the first slice. Anatomical and functional MRIs were acquired on a General Electrics Signa 1.5 tesla MRI scanner (GE, BUC, France). First, a high-resolution T1-weighted MRI scan (T1-MRI) was acquired with a three-dimensional inversion recovery spoiled gradient echo sequence (matrix size = 256×256×128; slice thickness = 1.5 mm). Second, a proton density/T2-weighted MRI scan (PD-MRI, T2-MRI) was acquired with 32 axial slices covering the entire brain and the superior part of the cerebellum (slice thickness = 3.8 mm). Finally, functional images were acquired with echo planar imaging blood oxygen level dependent (BOLD) sequence (repetition time = 6 s, echo time = 60 ms, flip angle = 90°, matrix size = 64×64×32, 50 volumes, 3.8-mm-thick slices) covering the same field of view as the T2-MRI acquisition.

### Construction of an old-adult template

Using voxel-based morphometry [65] (VBM5), each individual T1-MRIs of healthy elders were segmented according to the unified segmentation procedure [66] with spatial normalization included. Mean templates were calculated based on the individual segmented and normalized T1-MRIs, creating three separate old-adult templates according to tissue type (e.g. grey and white matters, cerebro-spinal fluid) which were then spatially smoothed using an 8-mm^3^ full width at half maximum (FWHM) Gaussian kernel.

### Functional image pre-processing

Functional images were processed and analyzed using the Statistical Parametric Mapping software (SPM5; Wellcome Trust Centre for Neuroimaging, London, United Kingdom; http://www.fil.ion.ucl.ac.uk/spml). The first six volumes of the functional acquisition were discarded, allowing for signal stabilization, and differences in slice acquisition timing were corrected. Images were realigned to correct for interscan movement with the creation of resliced mean functional volumes (mean-fMRI). For inter-modalities registration, rigid registration matrices (mean-fMRI onto T2-MRI, PD-MRI onto T1-MRI, T1-MRI onto the old-adult template) were computed, combined and then applied to fMRI volumes. Individual T1-MRIs were then segmented using the old-adult templates as priors (obtained previously, one for each tissue type; see above) and normalized. In order to set the fMRI volumes into our old-adult space, functional MRI images were resampled using the normalization parameters obtained in the segmentation step. Finally, data were spatially smoothed with an 8-mm^3^ FWHM Gaussian kernel.

### Data analyses

#### Behavioral data analysis

An adjusted t-test intended for single case analyses [67] was used for comparisons between each patient and healthy elders (for autobiographical scores and behavioral scales). Within-subject differences across past and future conditions were conducted using the modified t test [68] which assesses whether the difference between past and future periods in each patient differs significantly from the control group pattern. Results were considered significant at a conservative threshold of t>±2.576, p<0.01 to guard against Type 1 errors for multiple comparisons.

#### VBM analyses

In order to formally assess the extent of atrophy in each SD patient across the whole brain, we compared their structural MRI scans with those of the group of 12 healthy elders using voxel-based morphometry [66] (VBM5). Structural MRI images were analyzed using the optimised VBM procedure implemented in SPM5. Briefly, this involves a number of fully automated preprocessing steps including extraction of brain, spatial normalization into stereotactic (MNI) space, segmentation into grey and white matters and CSF compartments, correction for volume changes induced by spatial normalization (modulation), and smoothing with a 8-mm^3^ FWHM isotropic Gaussian kernel. The preprocessing procedures used in SPM5 have been shown to produce good results when matching brains with lesions to standardised templates [69]. We used the optional “modulation of non-linear effects only” which takes into account intracranial volume, hence controlling for differences in brain sizes. Analyses focussed on grey matter. Each patient's structural scan was compared to healthy elders' scans using a two sample t-test to investigate differences in grey matter volume. The significance level was set at p<0.05 FWE corrected for multiple comparisons, k>50 voxels (see Tables S2 to S5 in File S1).

#### fMRI data analyses

fMRI time series were modelled by a general linear model (GLM) including separate regressors for each of the experimental (past and future periods) and control conditions using SPM5. All regressors were convolved with the canonical hemodynamic response function (HRF). Data were high-pass filtered (cut-off period = 96 s). Coefficients for each regressor were estimated for each participant using maximum likelihood estimates to account for serial correlations in the data. At the first level, linear contrasts of the parameter estimates for each experimental regressor of interest were calculated for each participant, subtracting the corresponding control regressor (resulting in “period minus control task” contrasts). To examine the future thinking network in each patient at the individual level, t-statistic maps were generated for the following contrasts of interest: future minus control task and future minus past period (subtracting for each period the corresponding control regressor) for each participant. The second level random effects analysis was conducted over contrast images obtained at the first level. Each patient was compared to the group of healthy elders (i.e., patients were not grouped together) using the two sample t-test model of SPM5, as recommended by Henson's guidelines (2006; http://www.mrc-cbu.cam.ac.uk//personal/rik.henson/personal/Henson_Singlecase_06.pdf) and previously used to report single case fMRI data, notably in semantic dementia [70], [6]. Inter-group subtraction analyses were computed to determine which regions were differentially activated by each patient and healthy elders when comparing past and future periods. The significance level was set at p<0.05 FWE corrected for multiple comparisons, k>10 voxels. Coordinates of brain regions are reported in the MNI space. To control for the age difference between patients and healthy elders, age was included as covariate. EP's duration of education was higher compared to healthy elders. Since education and general intelligence are generally closely inter-related [71], variations due to differences in general intelligence between EP and healthy elders were controlled for by adding years of education as covariate for analyses comparing EP to healthy elders. Finally, to reveal pathophysiological properties of the future thinking brain network in each patient using a different method, plots of activation magnitude were obtained for each patient and average of healthy elders in several regions of interest (ROIs) of the conventional future thinking network (medial frontal gyrus, hippocampus, precuneus). For each participant, mean activation values corresponding to the difference in BOLD activation between the experimental (future period) and control tasks (for Figure S1: control task; for Figure S2: past period; see File S1), were extracted within each ROI using the “anatomical VOI analysis” of the fMRIroi SPM toolbox [54].

## Results

### Behavioral results

All patients were able to perform the tasks, both recalling past memories and envisioning future events, differences lay in the episodic nature of the events experienced. On the objective measures of episodicity, JPL and EP were able to pre-experience only one episodic future event (rated 3 or 4 on the episodic scale) and four semantic future events (rated 1 or 2). On the contrary, LL was able to pre-experience four episodic future events and one semantic future event. EG was able to pre-experience three episodic future events and two semantic future events. Of note, despite using verbal cues, all patients were all able to read and understand the cues.

Comparisons between each patient and healthy elders showed, on the objective measures of episodicity, significantly lower overall autobiographical score (AS) for the future and a trend for the strictly episodic score (ES) for the past for JPL (see Table 2). Based on the number of episodic events experienced, a trend showed that JPL and EP produced less episodic future events compared to healthy elders. On the subjective rating scales, main results indicated that emotional intensity was greater for the past and the future for EP compared to healthy elders. Concerning mental imagery, EP evoked a greater number of mental images for the future compared to healthy elders, although a trend showed that image quality of future mental images was less clear for EP compared to healthy elders. Within-subject comparisons, using the modified t test procedure [68], did not reveal statistical differences between past and future conditions for each patient (data not shown). Note that adding education as covariate in behavioral analyses for EP, following Crawford et al.'s [72] guidelines, did not change results (see Table S1 in File S1).

### VBM results

**Figure 1:**
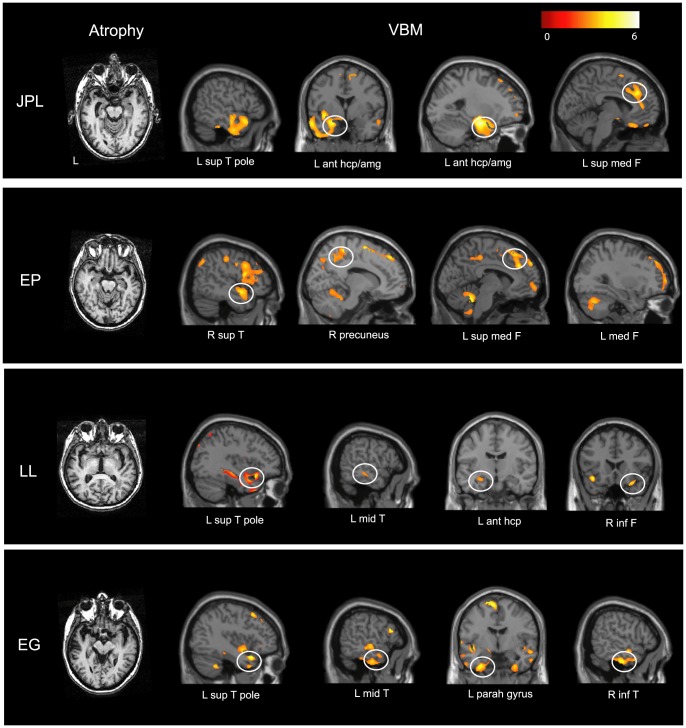
Structural brain scans of the four SD patients. Left panels show coronal sections through the brains of JPL, EP, LL and EG. Right panels show results of the VBM analysis superimposed on MRI scans for each patient at a corrected FWE threshold of p<0.05. The pronounced atrophy in superior medial frontal regions is apparent for JPL and EP and atrophy in left anterior hippocampus apparent for JPL and LL. Color scale shows voxel z-scores. See Tables S2 to S5 in File S1 for detailed VBM results. Abbreviations: amg = amygdala; ant = anterior; F = frontal; hcp = hippocampus; inf = inferior; L = left; med = medial; mid = middle; parah = parahippocampal; R = right; sup = superior; T = temporal.

Results depicted on Figure 1 and Tables S2 to S5 in File S1 reveal areas of grey matter volume loss in JPL, EP, LL and EG compared to healthy elders. All patients present lateral temporal atrophy, predominantly in its anterior portion (see Tables S2 to S5 in File S1). Of the four patients, three have predominant left-lateralized temporal atrophy (JPL, LL, EG) and EP has predominant right-lateralized temporal atrophy. JPL also presented atrophy in the left anterior hippocampus, left amygdala and frontal regions, in particular bilateral superior medial gyri.

Apart from predominantly right-lateralized temporal atrophy, EP also presented atrophy in frontal, in particular bilateral superior medial gyri and parietal regions (bilateral supramarginal, left inferior gyri) (see Table S3). EP showed preservation of anterior hippocampi.

Apart from predominantly left-lateralized temporal atrophy, LL also presented atrophy in left anterior hippocampus, left amygdala and bilateral inferior frontal gyri (see Table S4).

Apart from predominantly left-lateralized temporal atrophy, EG also presented atrophy in frontal regions (bilateral superior, left medial, bilateral inferior gyri) (see Table S5).

Strikingly, VBM analyses reveal that the two patients (JPL and EP) who have difficulties in envisioning the future in an episodic way present atrophy in bilateral superior medial frontal gyri which appears preserved in patients (LL and EG) who can project in the future in an episodic manner. Furthermore, the (left) anterior hippocampus, which is atrophied in JPL (and LL) may additionally explain his difficulties in episodic future projection (LL appears to compensate for this atrophy by hyperactivating the right anterior hippocampus, see below).

### fMRI results

#### Patient JPL

At the individual level, results for the contrast future minus control task for JPL showed an essentially left-lateralized network of regions comprising the left (inferior, middle, superior) frontal gyri, middle temporal gyrus, precuneus, occipital areas and cerebellum (see Table S6). Results for the contrast future minus past period for JPL did not reveal any activation at the given threshold (see Table S7).

**Figure 2:**
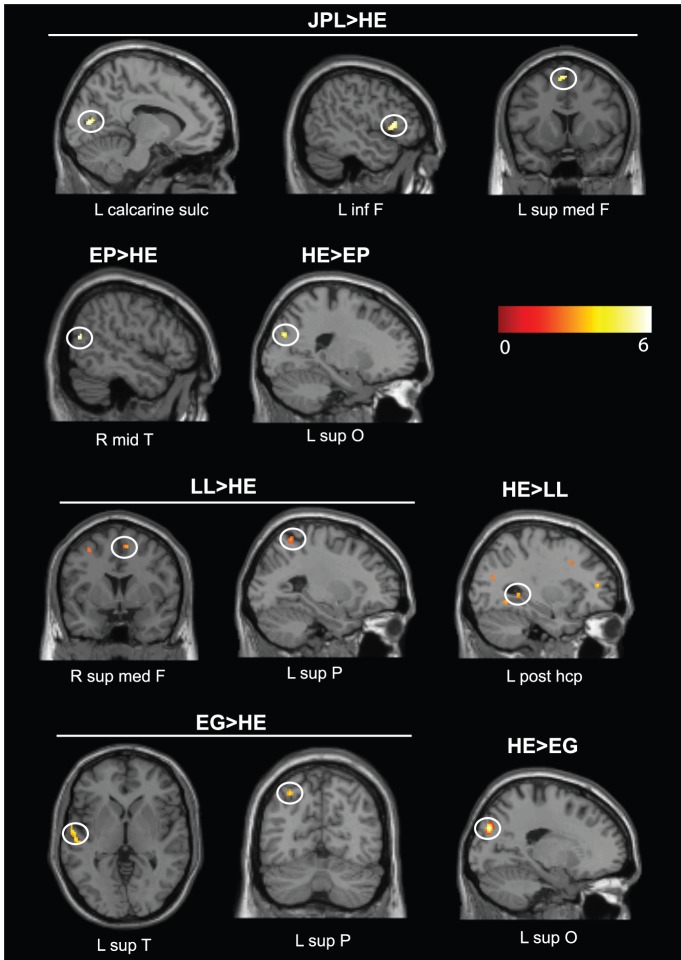
Results of the comparisons between the future period and the control task for EP, LL and EG depicting hyperactivations (Patient>HE) and lower activations (HE>Patient) compared to healthy elders (HE) at a corrected FWE threshold of p<0.05, k>10 voxels. Color scale shows voxel z-scores. See Table 4 for full details. Abbreviations: F = frontal; hcp = hippocampus; sup = superior; inf = inferior; L = left; med = medial; mid = middle; O = occipital; P = parietal; post = posterior; R = right; sulc = sulcus; T = temporal.

At the group level, the past period showed greater activations for JPL than healthy elders mainly in the left middle and inferior frontal gyri, bilateral cuneus and left insula compared to the control task (see Table 3). The future period showed greater activations for JPL than healthy elders mainly in the left calcarine sulcus, inferior and medial frontal gyri compared to the control task (see Table 4, Figure 2). Plots of activation magnitude confirm this medial frontal hyperactivation in JPL compared to healthy elders (see Figure S1). The past period showed greater activations for JPL, compared to healthy elders, in the right middle temporal gyrus compared to the future period (see Table 5). The future period showed no significant activations compared to the past period. No regions were less active in JPL compared to healthy elders.

**Table 3:** Results for the comparisons past>control task for JPL, EP, LL, EG compared to healthy elders (HE) with age and years of education (for EP) as covariates at a corrected FWE statistical threshold of p<0.05, k>10.

Regions	z-score	k	x	y	z
**JPL>HE**					
L inferior frontal gyrus	5.83	17	−34	16	28
L middle frontal gyrus	5.48	15	−44	16	40
R cuneus	5.78	21	14	−76	18
L cuneus	5.71	42	−10	−84	12
	5.40	16	−10	−72	22
L insula	5.66	24	−42	−4	−2
**HE>JPL**					
-	-	-	-	-	-
**EP>HE**					
L middle occipital gyrus	6.19	32	−46	−80	8
L precentral gyrus	5.59	47	−36	−14	46
**HE>EP**					
-	-	-	-	-	-
**LL>HE**					
R middle temporal gyrus	5.94	186	44	−58	8
L middle temporal gyrus	5.42	102	−40	−54	14
	5.13	102	−52	−58	18
L inferior frontal gyrus	5.86	67	−36	14	20
L middle frontal gyrus	5.40	67	−30	16	34
R cuneus	5.46	27	10	−86	28
L caudate	5.90	74	−16	26	2
**HE>LL**					
R anterior hippocampus	6.87	142	32	−4	−20
L parahippocampal gyrus	5.64	73	−30	−10	−24
R middle temporal gyrus	5.51	142	46	4	−20
R superior temporal pole	5.28	142	38	8	−20
L superior occipital gyrus	6.44	170	−20	−84	36
R superior occipital gyrus	5.66	20	26	−82	26
L middle occipital gyrus	5.37	170	−28	−76	28
	5.09	61	−24	−90	14
	5.41	19	−14	−88	−6
L cuneus	5.29	61	−10	−84	12
R inferior frontal gyrus	5.98	27	26	22	−18
R superior parietal gyrus	5.48	24	48	−38	58
R supramarginal gyrus	5.43	23	50	−34	38
	5.25	11	58	−36	30
R thalamus	5.98	81	16	−14	−2
L thalamus	5.19	64	−12	−14	−2
**EG>HE**					
L inferior frontal gyrus	6.66	52	−38	8	20
R middle cingulate gyrus	6.26	42	18	8	34
	5.76	29	16	−12	42
L anterior hippocampus	5.35	34	−30	−10	−22
R superior temporal pole	5.44	19	44	8	−22
**HE>EG**					
-	-	-	-	-	-

L: left, R: right.

**Table 4:** Results for the comparisons future>control task for JPL, EP, LL, EG compared to healthy elders (HE) with age and years of education (for EP) as covariates at a corrected FWE statistical threshold of p<0.05, k>10.

Regions	z-score	k	x	y	z
**JPL>HE**					
L calcarine sulcus	5.56	51	−10	−80	10
L inferior frontal gyrus	5.50	53	−48	20	8
L medial frontal gyrus	5.35	28	−4	10	62
**HE>JPL**					
**-**	-	-	-	-	-
**EP>HE**					
R middle temporal gyrus	5.35	10	52	−70	16
**HE>EP**					
L superior occipital gyrus	5.82	34	−20	−80	28
	5.39	34	−16	−76	36
R insula	5.36	29	36	−4	4
**LL>HE**					
R supramarginal gyrus	6.57	126	52	−36	38
	5.84	126	60	−36	28
	5.73	18	54	−24	32
L superior parietal gyrus	5.59	19	−26	−54	62
R medial frontal gyrus	5.66	15	10	4	56
	5.66	29	6	−14	66
	5.55	29	12	−10	70
L middle frontal gyrus	5.45	20	−34	6	54
R thalamus	5.78	13	8	−8	−8
**HE>LL**					
R superior frontal gyrus	5.81	233	26	4	60
L superior frontal gyrus	5.35	19	−20	4	62
	5.11	10	−16	10	58
R middle frontal gyrus	6.40	115	34	40	18
	5.64	115	26	42	22
	5.60	37	32	30	40
L middle frontal gyrus	5.77	25	−32	44	12
	5.41	54	−24	14	42
	5.33	54	−34	18	34
	5.15	54	−36	10	34
R inferior frontal gyrus	5.21	29	34	18	32
R supplementary motor area	5.31	50	8	−22	56
	5.71	31	4	−10	60
	5.28	16	14	18	58
L inferior parietal gyrus	5.79	55	−50	−24	38
R superior parietal gyrus	5.30	16	30	−66	50
L middle cingulate gyrus	5.77	50	0	−30	50
R fusiform gyrus	5.66	18	36	−42	−24
R inferior temporal gyrus	5.65	12	62	−40	−12
R superior temporal gyrus	5.55	22	56	−36	10
L posterior hippocampus	5.49	23	−30	−44	0
L posterior hippocampus	5.20	10	−24	−36	4
R precuneus	5.34	14	12	−54	66
L cuneus	5.82	36	−8	−76	18
R lingual gyrus	5.76	93	30	−48	−6
L middle occipital gyrus	5.15	10	−30	−72	20
R middle occipital gyrus	5.11	10	34	−76	26
R insula	5.97	54	34	−18	18
L caudate	6.04	102	−10	12	−8
	5.73	102	−4	6	−8
R caudate	5.17	102	8	10	−10
	5.91	58	14	22	10
R thalamus	5.55	19	2	−14	4
	5.45	27	12	−34	4
R vermis	5.64	36	6	−58	−16
**EG>HE**					
L superior temporal gyrus	5.64	45	−58	−10	0
	5.60	45	−56	−18	0
L superior parietal gyrus	5.48	16	−30	−66	52
**HE>EG**					
L superior occipital gyrus	6.96	118	−18	−82	54

L: left, R: right.

**Table 5:** Results for contrast past>future for JPL, EP, LL, EG compared to healthy elders (HE) with age and years of education (for EP) as covariates at a corrected FWE statistical threshold of p<0.05, k>10.

Regions	z-score	k	x	y	z
**JPL>HE**					
R middle temporal gyrus	5.62	14	44	−70	14
**EP>HE**					
L middle frontal gyrus	5.83	52	−34	−6	44
**LL>HE**					
R superior medial frontal gyrus	6.30	65	10	62	26
	5.46	83	4	66	4
L superior medial frontal gyrus	5.53	142	−4	64	28
	5.15	142	−6	62	20
	5.43	83	−8	62	0
L superior frontal gyrus	5.73	151	−16	58	16
	5.27	151	−20	60	24
	5.48	12	−18	36	52
	5.04	39	−20	48	16
L medial frontal gyrus	5.16	33	−12	50	−6
R medial frontal gyrus	5.41	12	2	−20	56
L middle frontal gyrus	6.19	161	−30	16	36
	6.11	151	−36	58	14
	6.08	52	−30	44	10
	5.45	29	−46	30	30
	5.40	39	−20	48	26
R middle frontal gyrus	6.00	34	32	16	54
	5.99	162	42	14	42
	5.69	34	32	10	48
L inferior frontal gyrus	5.15	17	−56	18	30
	5.26	12	−46	28	16
L posterior hippocampus	6.07	108	−26	−40	−2
	5.83	108	−24	−38	6
R parahippocampal gyrus	5.82	308	36	−38	−12
R middle temporal gyrus	5.66	51	54	−54	8
	5.23	51	44	−56	10
	5.39	42	18	−74	58
R superior temporal gyrus	5.56	40	58	−34	10
L inferior temporal gyrus	5.35	21	46	−68	8
	5.29	12	−46	−60	−12
R fusiform gyrus	5.34	35	26	−78	−6
R cuneus	6.08	59	12	−88	30
L cuneus	5.72	21	−10	−78	20
L middle occipital gyrus	5.98	48	−36	−74	24
	5.35	55	−24	−58	32
	5.14	80	−38	−78	36
R inferior occipital gyrus	5.43	35	34	−86	−4
	5.15	12	38	−76	−4
R angular gyrus	6.55	180	40	−62	40
	6.01	180	46	−72	36
L angular gyrus	5.57	55	−34	−54	36
L inferior parietal gyrus	5.67	60	−52	−24	38
	5.51	80	−32	−78	42
R inferior parietal gyrus	5.39	39	56	−52	46
	5.19	39	56	−44	44
R supramarginal gyrus	5.55	19	52	−22	22
R precuneus	5.74	35	12	−50	44
	5.48	13	8	−56	56
L precuneus	5.62	66	−12	−56	40
L anterior cingulate gyrus	5.43	20	−6	44	6
L middle cingulate gyrus	5.76	58	−4	−42	46
R thalamus	5.64	29	12	−32	4
	5.44	20	18	−20	14
R cerebellum	6.44	205	12	−62	−16
	5.75	205	18	−64	−22
	5.53	13	34	−42	−26
**EG>HE**					
L superior occipital gyrus	6.29	28	−16	−84	34

No regions were less active by patients compared to healthy elders for this contrast. L: left, R: right.

#### Patient EP

At the individual level, results for the contrast future minus control task for EP showed activation in the right precuneus (see Table S6). No activation was detected for the contrast future minus past period (see Table S7).

At the group level, the past period showed greater activations for EP than healthy elders in the left middle occipital and precental gyri compared to the control task (see Table 3). No regions were less active in EP compared to healthy elders for this contrast.

The future period showed greater activations for EP than healthy elders in the right middle temporal gyrus compared to the control task (see Table 4, Figure 2). EP showed less activation than healthy elders in the left superior occipital gyrus and right insula for the future period compared to the control task (see Table 4, Figure 2).

The past period revealed greater activations for EP than healthy elders in left middle frontal gyrus compared to the future period (see Table 5). No regions were less active in EP compared to healthy elders for this contrast.

**Figure 3:**
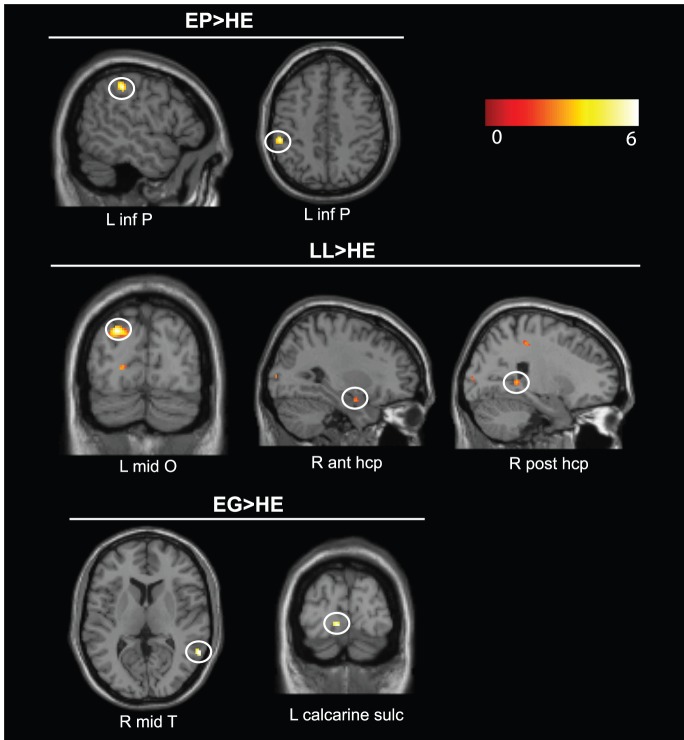
Results of the comparisons between the future and past periods for JPL, EP, LL and EG compared to healthy elders (HE) at a corrected FWE threshold of p<0.05, k>10 voxels. See Table 6 for full details. Abbreviations: ant = anterior; hcp = hippocampus; inf = inferior; L = left; mid = middle; O = occipital; P = parietal; post = posterior; R = right; sulc = sulcus; T = temporal.

Conversely, the future period showed greater activations in EP than healthy elders in the left parietal gyrus compared to the past period (see Table 6, Figure 3). No regions were less active in EP compared to healthy elders for this contrast.

**Table 6:** Results for contrast future>past for JPL, EP, LL, EG compared to healthy elders (HE) with age and years of education (for EP) as covariates at a corrected FWE statistical threshold of p<0.05, k>10.

Regions	z-score	k	x	y	z
**JPL>HE**					
-	**-**	**-**	**-**	**-**	**-**
**EP>HE**					
L inferior parietal cortex	6.90	91	−52	−34	46
	6.54	91	−44	−42	52
**LL>HE**					
L middle occipital gyrus	6.77	130	−22	−82	36
R middle occipital gyrus	5.72	12	26	−92	6
R supramarginal gyrus	6.68	262	52	−36	38
	6.28	262	60	−36	30
	6.11	262	54	−22	32
L superior parietal gyrus	5.44	15	−24	−54	60
R posterior hippocampus	5.80	23	24	−44	4
R anterior hippocampus	5.42	21	30	−4	−20
R middle temporal pole	5.28	15	46	4	−18
**EG>HE**					
R middle temporal gyrus	5.52	21	60	−52	8
L calcarine sulcus	5.35	14	−6	−90	−6

No regions were less active by patients compared to healthy elders for this contrast. L: left, R: right.

#### Patient LL

At the individual level, results for the contrast future minus control task for LL showed activation in the left supplementary motor area (see Table S6). Compared to the past period, the future period showed hyperactivation in the right middle occipital and supramarginal gyri (see Table S7).

At the group level, the past period showed greater activations for LL than healthy elders mainly in the bilateral middle temporal gyri, left middle and inferior frontal gyri, right cuneus and left caudate compared to the control task (see Table 3). LL showed less activation than healthy elders in essentially the right anterior hippocampus, left parahippocampal gyrus, lateral temporal and occipital gyri for the past period compared to the control task (see Table 3).

The future period revealed greater activations for LL than healthy elders mainly in the right supramarginal and left superior parietal gyri, right medial and left middle frontal gyri and right thalamus compared to the control task (see Table 4, Figure 2). LL showed less activation than healthy elders in essentially the left posterior hippocampus, frontal (bilateral superior, middle and right inferior) gyri, right precuneus, lateral parietal gyrus, right lateral temporal and occipital regions for the future period compared to the control task (see Table 4, Figure 2). Plots of activation magnitude confirm the significant lower activation in the left posterior hippocampus and right precuneus in LL compared to healthy elders (see Figure S1).

The past period showed greater activations for LL than healthy elders in the left posterior hippocampus, frontal (bilateral superior medial, middle, left superior and inferior frontal gyri), temporal (right middle, superior and left inferior temporal gyri), occipital (bilateral cuneus, middle and inferior occipital gyri) and parietal regions (angular, inferior parietal and supramarginal gyri, bilateral precuneus) compared to the future period (see Table 5). No regions were less active in LL compared to healthy elders for this contrast.

Conversely, the future period showed greater activations for LL than healthy elders in right anterior hippocampus, bilateral middle occipital gyri, right middle temporal pole and parietal regions (supramarginal and superior parietal gyri) compared to the past period (see Table 6, Figure 3). Plots of activation magnitude confirm this right anterior hippocampal hyperactivation in LL compared to healthy elders (see Figure S2). No regions were less active in LL compared to healthy elders for this contrast.

#### Patient EG

At the individual level, results for the contrast future minus control task for EG showed an essentially left-lateralized network of regions comprising the left middle frontal and temporal gyri (see Table S6). No activation was detected for the contrast future minus past period (see Table S7).

At the group level, the past period showed greater activations for EG than healthy elders in the left anterior hippocampus, left inferior frontal gyrus, right middle cingulate cortex and superior temporal pole compared to the control task (see Table 3). No regions were less active in EG compared to healthy elders for this contrast.

The future period showed greater activations for EG than healthy elders in the left superior temporal and superior parietal gyri compared to the control task (see Table 4, Figure 2). EG showed less activation than healthy elders in the left superior occipital gyrus for the future period compared to the control task (see Table 4, Figure 2).

The past period showed greater activations for EG than healthy elders in the left superior occipital gyrus compared to the future period (see Table 5). No regions were less active in EG compared to healthy elders for this contrast.

Conversely, the future period showed greater activations for EG than healthy elders in the right middle temporal gyrus and left calcarine sulcus compared to the past period (see Table 6, Figure 3). No regions were less active in EG compared to healthy elders for this contrast.

For sake of clarity, we also added results of the contrast future minus control task in healthy elders (see Table S8 in File S1). Results for the contrast future minus past period in healthy elders have been published previously [19].

## Discussion

**Figure 4:**
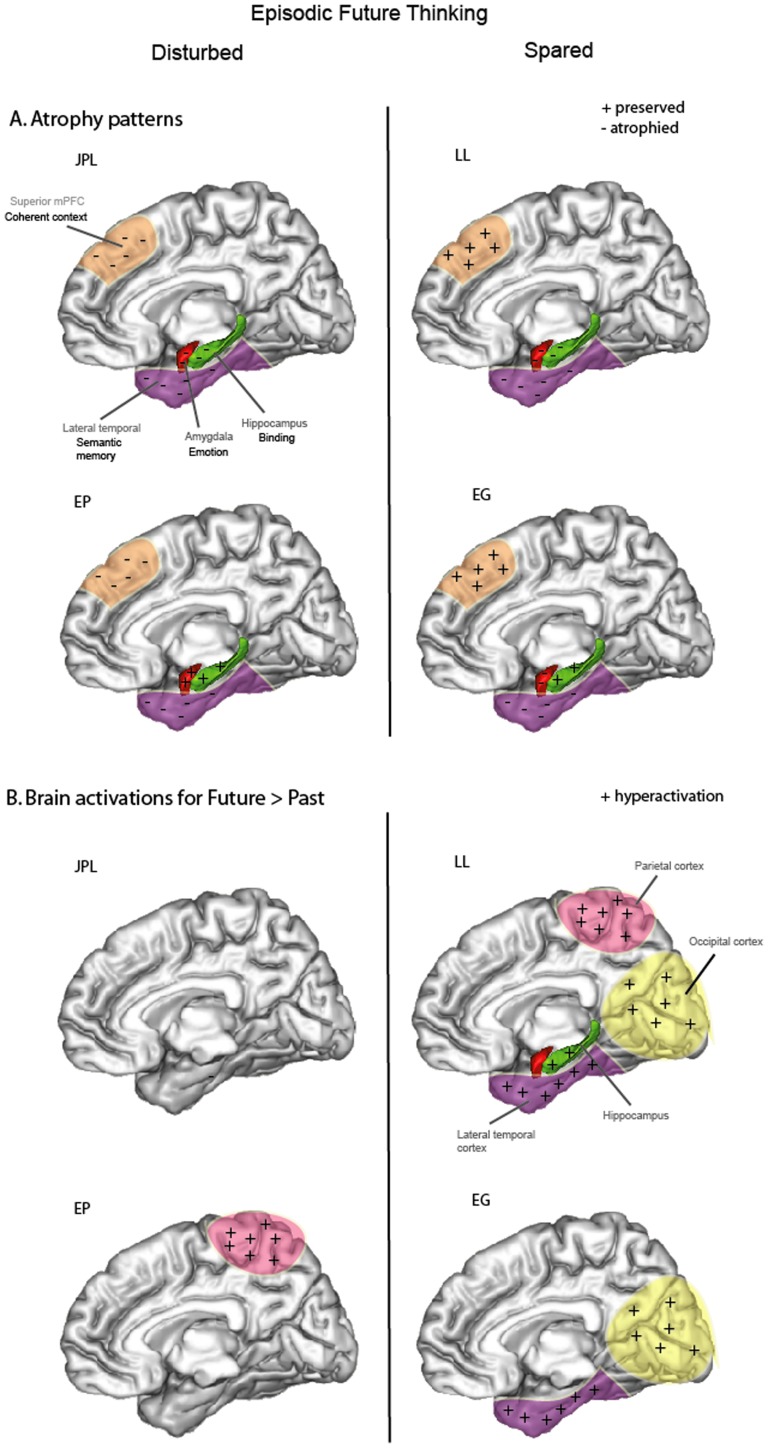
Summary representations of patients' ability to engage in episodic future thinking (left: disturbed; right: spared) depicting (A) localization of preserved (+) or atrophied (−) brain regions and associated hypothesized function (superior medial prefrontal cortex (mPFC): coherent context processing; hippocampus: binding; amygdala: emotional processing; lateral temporal cortex: semantic processing) and (B) brain activations for each patient compared to healthy elders for the contrast Future>Past thinking (see results on Table 6).

This work is, to our knowledge, the first fMRI study examining future projection in semantic dementia. While the sparse behavioral studies found that future projection was consistently impaired in SD [18], [16], [17], here we show that the capability of patients to project into their future largely depends on the structural integrity of certain brain regions, in particular the superior medial frontal cortex and anterior hippocampus. JPL presented atrophy in bilateral superior medial frontal gyri and left anterior hippocampus and had difficulties in experiencing episodic future events and to a certain extent past episodic events. Hyperactivations of neocortical (frontal and occipital) regions appeared inefficient in compensating for his deficit. EP presented atrophy in bilateral superior medial frontal gyri and, like JPL, could pre-experience only one episodic future event, but past episodic remembering was spared. However, behavioral ratings for the future were higher than those of healthy elders (in terms of emotion and mental imagery), suggesting that EP may have overestimated her capacities to project into the future. On the contrary, LL was able to pre-experience episodic future events. Although she had left anterior hippocampal atrophy, hyperactivation of its right counterpart during future compared to past thinking compensated efficiently for this atrophy. Finally, EG who presented integrity of superior medial frontal gyri and anterior hippocampi was also able to pre-experience episodic future events. Figure 4 depicts summary representations of patients' ability to engage in episodic future thinking (disturbed or spared) depicting sites of atrophy with associated hypothesized function and brain activations for each patient compared to healthy elders. Overall, VBM analyses showed that patients who had difficulties in envisioning the future in an episodic way (JPL and EP) presented specific atrophy in superior medial prefrontal cortex, while this region was relatively preserved in LL and EG who could engage in episodic future projection. Furthermore, JPL and LL presented atrophy in (left) anterior hippocampus, a region known to be crucial for episodic past and future thinking, but LL was able to compensate efficiently by hyperactivation of its right counterpart, while JPL could not. We will mainly focus on these two key regions in the following discussion, distinguishing patients who cannot (JPL and EP) or can (LL and EG) engage in episodic future thinking.

### Patients impaired in episodic future projection

JPL presented atrophy mainly in left anterior hippocampus, lateral (middle and inferior) temporal and frontal cortices, including in the bilateral superior medial frontal gyri. Behavioral ratings showed that he was impaired at episodic future projection and past remembering. The overall autobiographical score (AS) for the future and a trend for the strictly episodic autobiographical score (ES) for the past were significantly lower compared to healthy elders, indicating that JPL had difficulties in providing episodic details for future and past periods. A trend also showed that he produced less episodic future events compared to healthy elders. Irish et al. [16] showed that the future thinking deficit in SD was driven by a difficulty to provide “internal” details (i.e., episodic details, central to the event), while “external” details (i.e., semantic facts) were more numerous. With JPL, we confirm behavioral studies showing that SD patients are impaired at projecting into the future in an episodic way [18], [16], [17] and provide the neural correlates of these cognitive findings. JPL presented atrophy in bilateral superior medial frontal gyri (approximately corresponding to Brodmann areas 8 and 9). This area has been previously reported in episodic prospection studies [73], [74], [75], [36] and has a role in processing coherent contexts [36] (BA9), manipulating processes in working memory [76] (BA8), creative story generation [77] (BA8) and autonoetic consciousness [8], [37], essential to pre-experience episodic future events. Bilateral atrophy in this region may also have been responsible for JPL's inability to project into the future. JPL also presents atrophy in the left anterior hippocampus. We previously showed that anterior hippocampal integrity in SD is crucial for episodic past remembering [6], [70]. Here, we expand these findings to episodic future thinking which also requires anterior hippocampal integrity. The anterior hippocampus supports relational processing [31], [32], [33], including flexible recombination of details for past and future event construction [34]. Addis and Schacter [35] showed that future-associated activity in the anterior hippocampus was associated with higher demands on recombination of details. Left atrophy in this region may be in part responsible for JPL's inability to project into the future in an episodic way. Thus, we confirm that atrophy in crucial regions of the future thinking network (e.g., superior medial frontal gyrus and anterior hippocampus), overlapping with the autobiographical memory network, impairs episodic future projection. Examination of activation patterns at the individual level indicated that JPL failed to activate the core episodic future thinking network observed in healthy elders (reported in Table S8 and Viard et al. [19]), although several regions may show similar activation patterns across patient and healthy elders (e.g., precuneus, middle cingulate gyrus). Compared to healthy elders, JPL hyperactivated left (medial and inferior) frontal and occipital regions during future thinking compared to the control task. Plots of activation magnitude confirmed hyperactivation in the medial frontal gyrus in JPL compared to healthy elders. Yet, these hyperactivations did not efficiently compensate for atrophied regions, since he was unable to pre-experience episodic future events, as shown by behavioral results.

EP also presented atrophy in frontal cortices, including bilateral superior medial frontal gyri, and lateral (middle and superior) temporal cortices, but sparing of anterior hippocampi. EP was able to pre-experience only one episodic future event, most being semantic. Indeed, like JPL, a trend showed that EP produced less episodic future events compared to healthy elders. She was however not impaired at episodic past remembering. It appears that integrity of anterior hippocampi may have permitted EP to remember episodic past memories, but atrophy of bilateral superior medial frontal gyri may have been in part responsible for EP's deficit in episodic future thinking. Examination of activation patterns at the individual level indicated that, except for the right precuneus, EP failed to activate the core future thinking network observed in healthy elders (Table S8; [19]). Direct comparisons with healthy elders showed that EP hyperactived the inferior parietal and middle temporal cortices for the future compared to the past period, or to the control task, which appeared inefficient in compensating for her deficit, since she could pre-experience only one episodic future event.

EP had higher ratings on the scale of emotional intensity compared to healthy elders for the past and the future. Conversely, Irish et al. [78] found impaired emotion processing in a group of SD patients during past remembering and suspected that atrophy in the amygdala, generally reported in such patients, might explain their emotional processing deficit. Here, EP's bilateral amygdala were not atrophied compared to healthy elders, but she showed less activation than healthy elders in the right insula for the future compared to the control task, a region known to be implicated in emotional processing [79], [80], [81]. Hence, EP seems to evaluate the phenomenological nature of her mental evocations inadequately and may tend to exaggerate or overestimate her emotions on behavioral self-ratings. This may be paralleled with Irish et al.'s [16] findings of a disconnect between “objective” task performances (as shown here by scores on the episodic scale) and “subjective” phenomenological pre-experiencing (as shown here by self-ratings) when generating future events in SD. An early report by Snowden et al. [82] had already indicated that SD patients showed “exaggerated reactions to sensory stimuli” which fits with the present results.

Decreased activity in the occipital cortex for the future period compared to the control task was observed for EP compared to healthy elders. This decreased activity in a visuospatial area for the future contradicts EP's behavioral self-ratings on visual imagery which indicate a greater number of mental images for the future for EP compared to healthy elders. Yet, a trend showed that EP's image quality of future events was less clear compared to healthy elders. Overall, as for ratings of emotional intensity, EP may have overestimated the number of images she visualized.

Overall, JPL and EP both presented atrophy in bilateral superior medial prefrontal cortices, in addition to their lateral temporal atrophy, and were impaired at projecting into the future in an episodic way. JPL, unlike EP, was also impaired at remembering the past and presented atrophy in left anterior hippocampus. Thus, joint atrophy in bilateral superior medial prefrontal cortices and anterior hippocampi may have prevented episodic thinking in the past and the future (as observed in JPL). Atrophy in bilateral superior medial prefrontal cortices, but preservation of anterior hippocampal structures, appear to preserve past episodic recollection, at least to a certain extent, but impairs episodic future thinking (as observed in EP).

### Patients capable to engage in episodic future thinking

LL presented atrophy mainly in left anterior hippocampus, frontal (superior, inferior) and lateral (middle and superior) temporal cortices. LL was able to pre-experience episodic future events. Examination of activation patterns at the individual level showed that LL failed to activate the core future thinking network observed in healthy elders (Table S8; [19]), although several regions (e.g., occipital and parietal cortices) showed similar activation patterns across her and healthy elders. Nevertheless, she was able to engage in episodic future thinking and pre-experience future plans. Thus, at the neural level, she was able to develop different strategies from those of healthy elders which proved successful at the behavioral level since she was able to perform the future thinking task by envisionning several episodic future events. Indeed, hyperactivation in the right anterior hippocampus was detected for future compared to past thinking, confirmed by plots of activation magnitude. This right anterior hippocampal activation probably compensated for atrophy in its left counterpart. Addis et al. [74] showed that future event construction uniquely engaged the right hippocampus, possibly as a response to the novelty of these events. Beyond its role in relational processing in memory [83], this structure also has the capacity to bind disparate event details for novel future scenarios. Of note, out of the four patients, LL was the one who showed the most reductions in the pattern of neural activity compared to healthy adults, essentially in occipital regions for the past and occipital and frontal regions for the future. These reductions, however, appeared to be sufficiently compensated by hyperactivations in LL (in occipital cortex for the past and frontal cortex for the future) compared to healthy adults, since she was able to efficiently engage in episodic past and future thinking.

EG was able to engage in episodic past and future thinking in spite of atrophy in lateral temporal areas (superior, middle, inferior) gyri and poles and frontal (superior, middle) regions. Irish et al. [17] showed that atrophy in lateral temporal (inferior temporal cortex and temporal pole) correlated with deficits in episodic future thinking in their group of SD patients. Here, although correlations were not possible since we report single cases, we show that EG and LL, who were both able to engage in episodic future thinking, hyperactivated remnant tissue in lateral temporal lobes for the future compared to the past. Hence, our results corroborate Irish et al.'s [17] findings which suggest that lateral temporal cortex, strongly implicated in semantic memory, is critical for the construction of novel future events. Altogether, we extend Irish et al.'s [17] findings by showing that lateral temporal hyperactivation in addition to the integrity of superior medial frontal cortex and anterior hippocampus promote episodic future thinking in SD patients.

Overall, two patients out of four (LL and EG) were able to engage in episodic future thinking, unlike what has been reported in the behavioral literature [16], [17], [18]. Methodological differences may explain these discrepancies. Here, we used highly personalized cues derived from an interview with the spouse. These personally-relevant stimuli directly point to a specific event. Previous studies, instead, used single words which the patient should use to imagine a plausible future event. These stimuli may not immediately direct to a unique personally-relevant event which has already been planned by the participant. Hence, constructing personally-relevant sentences may be more helpful to trigger episodic future thinking in patients with semantic dementia who present integrity of superior medial prefrontal cortices and anterior hippocampus. Moreover, the group analysis approach used in previous studies may have obscured preservation of function that might have been evident in some (but not all) patients, which we highlighted here by using a case study approach.

### Limits

Albeit the clinical and theoretical relevance of the present findings, some potential limits should be mentioned. Given the rarity of patients with semantic dementia, we were not able to perfectly match each patient to the control group. Thus, results may have been influenced by differences in age and years of education (only EP) between each patient and the group of healthy elders. Yet, these variables were added as covariates in the analyses to attenuate any confound between patients and healthy elders. Adding a second covariate to control for differences in education between EP and healthy elders may have underestimated brain activations in EP. Yet, even with a higher education level compared to healthy elders, EP had difficulties in episodic future thinking. Hence, brain activations which may have been underestimated in the analyses (due to the addition of covariates) could not have been sufficient anyway to compensate for EP's deficit in episodic future thinking, since she was able to pre-experience only one episodic future event.

Another potential bias is that a male (JPL) was compared to a group of healthy females. Semantic dementia affects similarly males and females [84], [85] and no studies have shown a gender effect in this pathology. However, some studies have observed gender differences favouring women on episodic-memory tasks requiring verbal processing, but favouring men when requiring visuospatial processing [86], [87], [88]. Yet, the gender difference between JPL and the group of healthy elders may have played only a marginal role in the present results as mental retrieval of past memories and future projection involve both verbal and visuospatial processing.

Finally, unlike some studies in the future thinking field that use the cue-word technique with generic stimuli [16], [17], [74], the task used here was not to simulate or imagine a plausible fictitious future event, but to pre-experience an actual future event that was really supposed to happen in the next twelve months, prompted from a personally-relevant cue. In other words, future events had not happened yet, but were going to happen. Hence, since future events were actual projects that participants really intended to pursue in the future, these were planned in the past. Of note, in everyday life, this kind of future thinking is very frequent, probably the most frequent concerning the recent future. Thus, retrieval processes may have happened in the future thinking condition. Results from behavioral scales evaluating recent reactivation can indicate if each event was evoked recently or not. On the frequency of rehearsal scale, LL and EG obtained scores below 4.5 indicating that they did not frequently rehearse their future plans in the past. EP obtained a score of 9.5, suggesting that she frequently rehearsed her future plans. Yet, EP envisioned only one episodic future event (the four other events being semantic). Thus, she was probably able to envision this unique future plan in an episodic way because she had frequently rehearsed it. On the last recall scale, EP, LL and EG obtained scores indicating that, on average, they last recalled their future plans a sufficiently long time ago, minimizing a possible retrieval effect. It is nevertheless possible that by presenting cues referring to actual future plans, a retrospective thinking effect may have happened. However, unlike some future thinking studies in which participants are asked to imagine plausible future events (which are not necessarily going to happen) using generic cues, here we chose to study the self-projecting aspect of future thinking in which future actions are planned beforehand [12]. It would be interesting to study the purely imaginative aspect, as some studies have already explored [89].

## Conclusion

Altogether, this study aimed at examining the neural correlates of future projection in SD compared to past remembering. Main results indicated that episodic future projection in patients with lateral temporal atrophy largely depends on the integrity of superior medial prefrontal cortices and anterior hippocampi. With atrophy in the bilateral superior medial frontal gyri and left anterior hippocampus, JPL was unable to engage into past or future episodic thinking, in spite of neocortical hyperactivity which appeared inefficient in compensating for atrophy within these key regions. EP presented atrophy in bilateral superior medial frontal gyri and was, like JPL, able to pre-experience only one episodic future event, but past remembering appeared relatively preserved. On the contrary, LL despite atrophy in the left anterior hippocampus, was able to efficiently compensate for the deficit by hyperactivating its right counterpart during episodic future thinking. EG, who did not show atrophy in the superior medial frontal gyrus nor anterior hippocampus, was able to envision episodic future events. Altogether, these findings suggest that patients' ability to envision the future in an episodic way largely depends on the severity and localization of their atrophy, in addition to their lateral temporal atrophy. Our results indicate that the integrity of the superior medial frontal gyri and anterior hippocampi, in addition to the use of personally-relevant cues, are essential to trigger episodic future thinking.

## Supporting Information

File S1 Contains the following files: **Table S1**: Behavioral ratings comparing EP and healthy elders (HE) for past and future periods with years of education added as covariate. Significant results appear in bold (p<0.05). AS = autobiographical score; ES = strictly episodic autobiographical score; Nb = number; p = probability; sd = standard deviation. **Table S2**: Areas of grey matter volume loss in JPL vs. 12 healthy elders at a corrected FWE statistical threshold of p<0.05. L: left, R: right. **Table S3**: Areas of grey matter volume loss in EP vs. 12 healthy control participants at a corrected FWE statistical threshold of p<0.05. L: left, R: right. **Table S4**: Areas of grey matter volume loss in LL vs. 12 healthy elders at a corrected FWE statistical threshold of p<0.05. L: left, R: right. **Table S5**: Areas of grey matter volume loss in EG vs. 12 healthy control participants at a corrected FWE statistical threshold of p<0.05. L: left, R: right. **Table S6**: Results for the contrast future>control task for JPL, EP, LL and EG at a corrected FWE statistical threshold of p<0.05, k>10. **Table S7**: Results for the contrast future>past period for JPL, EP, LL and EG at a corrected FWE statistical threshold of p<0.05, k>10. L: left, R: right. **Table S8**: Results for the contrast future>control task for healthy elders at a corrected FWE statistical threshold of p<0.05, k>10. L: left, R: right. SMA = supplemental motor area. **Figure S1**: plots of activation magnitude for each patient (JPL, EP, LL and EG) and average of healthy elders (Ctl) in the bilateral medial frontal gyri, hippocampi and precuneus for the contrast future>control task. **Table S9**: Brief explanation of the neuropsychological tests used to test participants and what they measure. **Table S10**: Specificity scoring chart of past and future episodic tasks, performed at debriefing.
(DOC)
