# Supplementary material for: Episodic Future Thinking in Semantic Dementia: A Cognitive and fMRI Study
Source: PLoS One. 2014 Oct 21;9(10):e111046. doi: 10.1371/journal.pone.0111046 (PMC4205092; doi:10.1371/journal.pone.0111046)
Supplement: File S1 — Contains the following files: Table S1: Behavioral ratings comparing EP and healthy elders (HE) for past and future periods with years of education added as covariate. Significant results appear in bold (p<0.05). AS = autobiographical score; ES = strictly episodic autobiographical score; Nb = number; p = probability; sd = standard deviation. Table S2: Areas of grey matter volume loss in JPL vs. 12 healthy elders at a corrected FWE statistical threshold of p<0.05. L: left, R: right. Table S3: Areas of grey matter volume loss in EP vs. 12 healthy control participants at a corrected FWE statistical threshold of p<0.05. L: left, R: right. Table S4: Areas of grey matter volume loss in LL vs. 12 healthy elders at a corrected FWE statistical threshold of p<0.05. L: left, R: right. Table S5: Areas of grey matter volume loss in EG vs. 12 healthy control participants at a corrected FWE statistical threshold of p<0.05. L: left, R: right. Table S6: Results for the contrast future>control task for JPL, EP, LL and EG at a corrected FWE statistical threshold of p<0.05, k>10. Table S7: Results for the contrast future>past period for JPL, EP, LL and EG at a corrected FWE statistical threshold of p<0.05, k>10. L: left, R: right. Table S8: Results for the contrast future>control task for healthy elders at a corrected FWE statistical threshold of p<0.05, k>10. L: left, R: right. SMA = supplemental motor area. Figure S1: plots of activation magnitude for each patient (JPL, EP, LL and EG) and average of healthy elders (Ctl) in the bilateral medial frontal gyri, hippocampi and precuneus for the contrast future>control task. Table S9: Brief explanation of the neuropsychological tests used to test participants and what they measure. Table S10: Specificity scoring chart of past and future episodic tasks, performed at debriefing. (DOC) [file pone.0111046.s001.doc]

**Episodic future thinking in semantic dementia: a cognitive and fMRI study**

Viard et al.

**Supporting Information**

**Table S1:** Behavioral ratings comparing EP and healthy elders (HE) for past and future periods with years of education added as covariate. Significant results appear in bold (p<0.05). AS = autobiographical score; ES = strictly episodic autobiographical score; Nb=number; p=probability; sd = standard deviation.

|  | **HE** | | **EP** | |
| --- | --- | --- | --- | --- |
|  | **Past** | **Future** | **Past** | **Future** |
| **Episodic scores** | | | | |
| **AS score** | | | | |
| Mean | 3.73 (±0.41) | 3.17 (±0.57) | 3.60 | 2.40 |
| ± sd |
| p |  |  | 0.42 | 0.19 |
| **ES score** | | | | |
| Mean | 3.20 (±1.08) | 1.73 (±1.31) | 2.40 | 0.80 |
| ± sd |
| p |  |  | 0.31 | 0.32 |
| **Nb episodic events** | | | | |
| Mean | 4.75 | 4.00 (±1.16) | 5.00 | 1.00 |
| ± sd | (±0.45) |
| p |  |  | 0.36 | 0.05 |
| **Emotion** | | | | |
| **Intensity** | | | | |
| Mean | 5.10 (±1.59) | 4.80 (±1.72) | 9.50 | 9.50 |
| ± sd |
| p |  |  | **0.04** | **0.05** |
| **Valence** | | | | |
| Mean | 6.57 (±1.26) | 6.75 (±1.05) | 7.80 | 9.00 |
| ± sd |
| p |  |  | 0.26 | 0.09 |
| **Imagery** | | | | |
| **Mental strategy** | | | | |
| Mean | 8.65 (±1.83) | 8.86 (±1.69) | 10.00 | 10.00 |
| ± sd |
| p |  |  | 0.31 | 0.33 |
| **Image number** | | | | |
| Mean | 5.06 (±2.12) | 3.72 (±1.49) | 10.00 | 10.00 |
| ± sd |
| p |  |  | 0.07 | **0.008** |
| **Image quality** | | | | |
| Mean | 9.13 (±1.25) | 8.57 (±1.69) | 9.50 | 5.00 |
| ± sd |
| p |  |  | 0.42 | 0.09 |
| **Perspective** | | | | |
| Mean | 1.40 (±0.59) | 1.22 (±0.74) | 1.00 | 1.00 |
| ± sd |
| p |  |  | 0.33 | 0.42 |
| **Consciousness** | | | | |
| **R/K** |  |  |  |  |
| Mean  ±sd | 9.38 (±0.61) | 6.28 (±3.47) | 9.90 | 5.00 |
| p |  |  | 0.29 | 0.40 |
| **Repetition** | | | | |
| **Rehearsal frequency** | | | | |
| Mean | 5.51 (±1.99) | 4.39 (±2.02) | 0.00 | 9.50 |
| ± sd |
| p |  |  | **0.04** | 0.06 |
| **Last recall** | | | | |
| Mean | 1.61 (±0.89) | 1.46 (±1.49) | 0.50 | 1.00 |
| ± sd |
| p |  |  | 0.21 | 0.42 |

**Table S2:** Areas of grey matter volume loss in JPL vs. 12 healthy elders at a corrected FWE statistical threshold of p<0.05. L: left, R: right.

| **Regions** | **z score** | **x** | **y** | **z** |
| --- | --- | --- | --- | --- |
| L anterior hippocampus | 6.57 | -25 | -7 | -23 |
| L anterior hippocampus | 6.34 | -27 | -17 | -18 |
| L amygdala | 6.30 | -23 | -7 | -21 |
| L inferior temporal gyrus | 7.00 | -59 | -14 | -28 |
| L middle temporal gyrus | 6.24 | -58 | -1 | -23 |
| L superior temporal pole | 6.17 | -51 | 14 | -17 |
| L inferior temporal gyrus | 6.03 | -40 | -21 | -24 |
| L superior temporal gyrus | 5.34 | -37 | -8 | -11 |
| L middle temporal gyrus | 6.31 | -63 | -33 | 5 |
| L inferior temporal gyrus | 6.30 | -62 | -30 | -20 |
| R middle temporal gyrus | 6.28 | 53 | 9 | -28 |
| L middle temporal pole | 5.84 | -30 | 2 | -41 |
| R middle temporal gyrus | 5.79 | 59 | -9 | -27 |
| L superior medial frontal gyrus | 6.40 | -5 | 34 | 31 |
| R superior medial frontal gyrus | 6.35 | 5 | 23 | 39 |
| R superior medial frontal gyrus | 6.35 | 8 | 46 | 44 |
| L superior medial frontal gyrus | 6.10 | -3 | 18 | 42 |
| R superior frontal gyrus | 6.09 | 22 | 66 | 4 |
| R middle frontal gyrus | 5.97 | 28 | 46 | 36 |
| R inferior frontal gyrus | 5.96 | 22 | 15 | -23 |
| L superior frontal gyrus | 5.84 | -13 | 30 | -25 |
| L superior frontal gyrus | 5.76 | -14 | 16 | -22 |
| L inferior frontal gyrus | 5.74 | -54 | 24 | 11 |
| L superior frontal gyrus | 5.61 | -23 | 60 | -2 |
| L anterior cingulate gyrus | 5.71 | -5 | 42 | 9 |
| L insula | 6.35 | -36 | 1 | -6 |
| R supramarginal gyrus | 5.77 | 62 | -19 | 27 |

**Table S3** : Areas of grey matter volume loss in EP vs. 12 healthy control participants at a corrected FWE statistical threshold of p<0.05. L: left, R: right.

| **Regions** | **z score** | **x** | **y** | **z** |
| --- | --- | --- | --- | --- |
| L middle temporal gyrus | 5.70 | -63 | -33 | 5 |
| R superior temporal gyrus | 5.53 | 51 | 2 | -13 |
| R superior temporal gyrus | 5.34 | 52 | -5 | -7 |
| R middle temporal pole | 5.30 | 39 | 19 | -34 |
| R middle temporal gyrus | 5.29 | 42 | 7 | -24 |
| L superior medial frontal gyrus | 6.00 | -5 | 34 | 31 |
| L superior medial frontal gyrus | 5.78 | -9 | 39 | 51 |
| L superior medial frontal gyrus | 5.52 | -12 | 53 | -1 |
| R superior medial frontal gyrus | 5.58 | 46 | 13 | 34 |
| R superior frontal gyrus | 6.43 | 15 | 0 | 66 |
| R medial frontal gyrus | 5.80 | 7 | 4 | 52 |
| L medial frontal gyrus | 5.39 | -5 | 26 | 53 |
| L superior frontal gyrus | 5.89 | -13 | 23 | 58 |
| R superior frontal gyrus | 5.86 | 12 | 42 | 48 |
| R supramarginal gyrus | 6.55 | 64 | -35 | 28 |
| L supramarginal gyrus | 6.31 | -61 | -22 | 35 |
| L inferior parieral gyrus | 5.76 | -58 | -30 | 41 |
| R angular gyrus | 5.95 | 51 | -63 | 38 |
| R superior parietal gyrus | 5.63 | 22 | -72 | 47 |
| R precuneus | 5.88 | 5 | 69 | 39 |
| L precuneus | 5.73 | -11 | -40 | 55 |
| R caudate | 6.11 | 14 | 21 | 2 |
| L cerebellum | 6.06 | -43 | -52 | -36 |
| L cerebellum | 5.63 | -15 | -49 | -22 |

**Table S4** : Areas of grey matter volume loss in LL vs. 12 healthy elders at a corrected FWE statistical threshold of p<0.05. L: left, R: right.

| **Regions** | **z score** | **x** | **y** | **z** |
| --- | --- | --- | --- | --- |
| L anterior hippocampus | 5.51 | -24 | -8 | -21 |
| L amygdala | 6.15 | -29 | 2 | -19 |
| L middle temporal pole | 5.40 | -35 | 18 | -40 |
| L middle temporal gyrus | 5.33 | -56 | -26 | -9 |
| L middle temporal gyrus | 5.33 | -55 | -27 | -8 |
| R middle temporal pole | 5.32 | 38 | 18 | -37 |
| L superior temporal pole | 5.31 | -18 | 9 | -28 |
| R inferior frontal gyrus | 6.60 | 31 | 22 | -22 |
| L inferior frontal gyrus | 6.11 | -38 | 23 | -16 |
| R insula | 6.07 | 32 | 27 | 6 |
| R precuneus | 5.32 | 14 | -79 | 46 |

**Table S5** : Areas of grey matter volume loss in EG vs. 12 healthy control participants at a corrected FWE statistical threshold of p<0.05. L: left, R: right.

| **Regions** | **z score** | **x** | **y** | **z** |
| --- | --- | --- | --- | --- |
| L parahippocampal gyrus | 5.64 | -19 | -5 | -25 |
| L amygdala | 5.79 | -26 | 0 | -19 |
| R middle temporal gyrus | 6.99 | 53 | 9 | -29 |
| L inferior temporal gyrus | 6.82 | -59 | -14 | -28 |
| L inferior temporal gyrus | 5.35 | -52 | -6 | -34 |
| R inferior temporal gyrus | 6.67 | 56 | -16 | -33 |
| R middle temporal gyrus | 5.87 | 58 | -6 | -27 |
| L middle temporal gyrus | 6.44 | -58 | -1 | -24 |
| L middle temporal pole | 6.04 | -23 | 1 | -37 |
| L superior temporal pole | 6.24 | -39 | 16 | -27 |
| L superior temporal pole | 6.14 | -50 | 13 | -17 |
| L middle temporal pole | 6.08 | -29 | 18 | -32 |
| L inferior temporal gyrus | 6.13 | -62 | -38 | -16 |
| L middle temporal gyrus | 6.04 | -57 | -25 | -9 |
| L middle temporal gyrus | 5.83 | -56 | -13 | -7 |
| L superior temporal pole | 5.79 | -18 | 9 | -28 |
| R middle temporal pole | 5.91 | 39 | 13 | -39 |
| L middle temporal gyrus | 5.86 | -65 | -40 | -1 |
| R middle temporal gyrus | 5.80 | 59 | -5 | -16 |
| L superior temporal pole | 5.41 | -22 | 5 | -26 |
| L inferior temporal gyrus | 5.63 | -45 | 7 | -39 |
| L middle temporal gyrus | 5.38 | -51 | 5 | -32 |
| L medial frontal gyrus | 6.70 | -8 | -3 | 67 |
| L medial frontal gyrus | 6.65 | -4 | 3 | 63 |
| L superior frontal gyrus | 5.96 | -16 | -1 | 69 |
| L middle frontal gyrus | 6.44 | -37 | 16 | 51 |
| L inferior frontal gyrus | 6.09 | -25 | 9 | -21 |
| L inferior frontal gyrus | 5.39 | -16 | 13 | -21 |
| R inferior frontal gyrus | 5.64 | 52 | 29 | -6 |
| R superior frontal gyrus | 5.86 | 23 | 17 | 59 |
| R fusiform gyrus | 6.36 | 31 | -4 | -40 |
| L fusiform gyrus | 5.79 | -31 | -14 | -34 |
| L insula | 6.00 | -37 | 8 | -5 |
| L insula | 5.65 | -38 | 2 | -12 |
| L cerebellum | 5.97 | -40 | -49 | -41 |
| R cerebellum | 5.45 | 14 | -82 | -28 |

**Table S6:** Results for the contrast future>control task for JPL, EP, LL and EG at a corrected FWE statistical threshold of p<0.05, k>10.

| **Regions** | **z score** | **k** | **x** | **y** | **z** |
| --- | --- | --- | --- | --- | --- |
| **JPL** |  |  |  |  |  |
| L SMA | inf | 730 | -4 | 8 | 64 |
| L middle cingulate gyrus | 5.17 | - | -10 | 22 | 38 |
| L inferior frontal gyrus | 7.37 | 442 | -52 | 12 | 4 |
| L inferior frontal gyrus | 7.21 | - | -46 | 20 | 12 |
| L inferior frontal gyrus | 6.50 | - | -48 | 22 | 4 |
| L middle frontal gyrus | 5.24 | 191 | -40 | 16 | 50 |
| L middle frontal gyrus | 6.02 | 43 | -22 | 28 | 50 |
| L superior frontal gyrus | 5.33 | 31 | -10 | 64 | 22 |
| L middle temporal gyrus | 5.84 | 69 | -50 | -40 | 0 |
| L precuneus | 5.90 | 33 | -4 | -40 | 66 |
| L cuneus | 5.56 | 45 | -2 | -78 | 32 |
| L middle occipital gyrus | 5.52 | 32 | -40 | -78 | 30 |
| L fusiform gyrus | 5.28 | 11 | -42 | -52 | -26 |
| R cerebellum | 7.82 | 2041 | 34 | -68 | -24 |
| R cerebellum | 7.29 | - | 48 | -70 | -28 |
|  |  |  |  |  |  |
| **EP** |  |  |  |  |  |
| R precuneus | 5.65 | 25 | 2 | -34 | 64 |
|  |  |  |  |  |  |
| **LL** |  |  |  |  |  |
| L SMA | 5.81 | 51 | 0 | 6 | 58 |
|  |  |  |  |  |  |
| **EG** |  |  |  |  |  |
| L middle frontal gyrus | 6.39 | 142 | -46 | 0 | 52 |
| L middle temporal gyrus | 6.06 | 20 | -64 | -40 | 4 |
| L middle temporal gyrus | 5.47 | 11 | -60 | -50 | 6 |

*Abbreviations*: L: left, R: right; SMA=supplementary motor area.

**Table S7** : Results for the contrast future>past period for JPL, EP, LL and EG at a corrected FWE statistical threshold of p<0.05, k>10. L: left, R: right.

| **Regions** | **z score** | **k** | **x** | **y** | **z** |
| --- | --- | --- | --- | --- | --- |
| **JPL** |  |  |  |  |  |
| - | - |  | - | - | - |
|  |  |  |  |  |  |
| **EP** |  |  |  |  |  |
| - | - |  | - | - | - |
|  |  |  |  |  |  |
| **LL** |  |  |  |  |  |
| R middle occipital gyrus | 5.74 | 18 | 28 | -96 | 12 |
| R supramarginal gyrus | 5.38 | 50 | 58 | -40 | 30 |
|  |  |  |  |  |  |
| **EG** |  |  |  |  |  |
| - | - |  | - | - | - |

**Table S8** : Results for the contrast future>control task for healthy elders at a corrected FWE statistical threshold of p<0.05, k>10. L: left, R: right. SMA=supplemental motor area.

| **Regions** | **k** | **z score** | **x** | **y** | **z** |
| --- | --- | --- | --- | --- | --- |
| L SMA | 62 | 5.64 | 2 | 12 | 50 |
| R SMA | - | 5.49 | 4 | 4 | 60 |
| L superior frontal gyrus | 12 | 5.36 | -16 | 38 | 52 |
| L precuneus | 264 | 6.34 | -16 | -58 | 14 |
| L precuneus | 29 | 5.41 | 0 | -66 | 42 |
| R precuneus | 60 | 5.30 | 6 | -54 | 12 |
| L inferior parietal gyrus | 10 | 5.64 | -46 | -58 | 42 |
| L angular gyrus | 84 | 5.46 | -42 | -74 | 38 |
| L angular gyrus | - | 5.24 | -46 | -66 | 32 |
| R angular gyrus | 11 | 5.28 | 46 | -72 | 36 |
| L middle cingulate gyrus | 35 | 5.41 | -8 | -44 | 36 |
| R cerebellum | 136 | 7.04 | 16 | -78 | -16 |
| L cerebellum | - | 5.58 | -10 | -76 | -16 |

**Figure S1**: plots of activation magnitude for each patient (JPL, EP, LL and EG) and average of healthy elders (Ctl) in the bilateral medial frontal gyri, hippocampi and precuneus for the contrast future>control task.


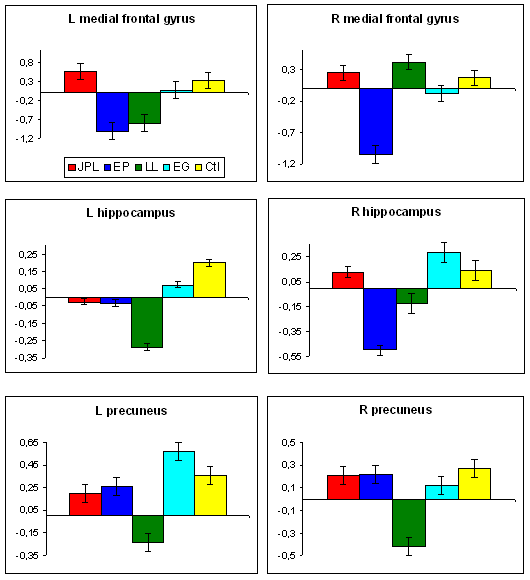


**Figure S2**: plots of activation magnitude for each patient (JPL, EP, LL and EG) and average of healthy elders (Ctl) in the bilateral medial frontal gyri, hippocampi and precuneus for the contrast future>past period.


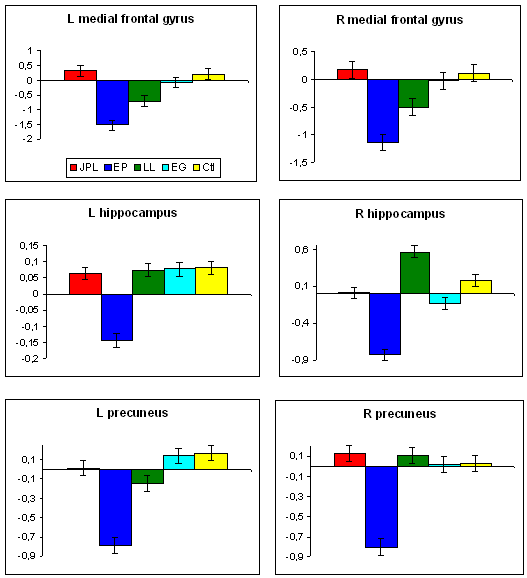


**Table S9**: Brief explanation of the neuropsychological tests used to test participants and what they measure.

1. The **Grober and Buschke** test was used to measure verbal episodic memory (Grober and Buschke, 1987). The first phase of this test consists in encoding 16 words belonging to 16 different semantic categories. Words are displayed 4-by-4 on a sheet and participants have to point to words in response to their semantic category. Every four words, an immediate cued recall is performed using semantic category cues, to ensure that encoding has taken place. If participants fail, the experimenter shows the sheet again so that all 16 items are retrieved at immediate cued recall. The encoding phase is followed by three recall trials (each comprising free recall and, if necessary, categorical cued recall). Between each trial, participants are asked to count backwards for 20 seconds. A recognition test is performed after the three recall trials. After 20 minutes, a free recall and delayed recall is performed. The maximum score for each recall is 16/16.
2. The **Semantic Knowledge Task** (Desgranges et al., 1996) is composed of 18 drawings (taken from the 260 pictures of Snodgrass and Vanderwart, 1980), divided into three categories (animals, plants, objects). Participants are first required to name the drawings (i.e., picture naming). If they fail, a recognition test of the noun is carried out: the correct noun and five others from the same semantic category are presented to the subject one after the other. Next, the participant is asked to answer a series of questions for each of the 18 items. The first concerns knowledge of the superordinate category ("Does it occur naturally or is it manmade?"). The second concerns category membership ("Is it an animal, a plant or an object?"). The third refers to the subcategory ("Is it a mammal, an insect or a bird?"). Finally, there are three questions concerning specific attributes: either functional ("Is it edible?") or perceptive ("Is its shape elongated?"). The score is the total number of correct answers, the maximum being 144 (8 questions for the 18 items).
3. The **BECS** **GRECO Semantic Battery** (Merck et al., 2011) is an explicit semantic memory test with a subtest, derived from the Pyramid and Palm Trees Test (Howard and Patterson, 1992), consisting in a forced-choice semantic matching task of 40 items (20 living and 20 non living) in verbal (words) and visual (black and white pictures) input modalities.
4. The **Mill Hill test** (French translation by Deltour 1993) is a multiple-choice synonym vocabulary questionnaire which assesses verbal knowledge.
5. In the word **fluency tasks** (Cardebat et al., 1990), the participant is asked to produce in 2 min as many words as possible which comply to a certain category (names of animals, i.e. semantic fluency) or orthographic (words beginning with letter p, i.e. formal fluency).
6. For picture naming, we selected the 80 drawings taken from the **LEXIS battery** (de Partz, 2001) representing natural (n=32) or manufactured objects (n=48). Each item’s name is characterized by its lexical frequency and length in syllables.
7. The **Mattis dementia rating scale** (Mattis, 1976) examines 5 areas that are particularly sensitive to the behavioral changes that characterize senile dementia of the Alzheimer’s type. Five areas are covered: (1) attention (digits forward and backward up to four; followed by two consecutive demands, e.g. “Open your mouth and close your eyes”); (2) initiation and perseveration (e.g., name articles in a supermarket, repeat series of one-syllable rhymes; perform double alternating hand movements, copy a row of alternating O’s and X’s); (3) construction (e.g., copy a diamond in a square, copy a set of parallel lines, write name); (4) conceptual (e.g., four WAIS-type Similarities items, identify which of three items is different); and (4) memory (e.g., delayed recall of five-word sentences, personal orientation, design recall). A scoring system permits test-retest comparisons of both individual subscales and a total score (total score/144).
8. The **French celebrities questionnaire** (Belliard et al., 2001) was constructed for SD patients. It is composed of a familiarity decision subtest and a questionnaire based on recognition tasks (dead/alive, nationality, profession, specific knowledge) concerning 10 celebrities (e.g., J.P. Belmondo, A. Delon), most of them still alive, presented by name and photograph. The first step consists in a familiarity decision task concerning famous people presented by name. The second step is a questionnaire concerning the names classified as familiar.

**Table S10**: Specificity scoring chart of past and future episodic tasks, performed at debriefing.

4 Specific event (isolated, situated in time and space) with details (unfolding of actions, thoughts, perceptions, images, etc)

3 Specific event (isolated, situated in time and space) with few details

2 Generic event (repeated or prolonged over time, situated in time and space)

1 Vague event (repeated or prolonged over time, not situated in time and space)

0 Absence of memory or general information about a theme
